# A bacterial pigment provides cross-species protection from H_2_O_2_- and neutrophil-mediated killing

**DOI:** 10.1073/pnas.2312334121

**Published:** 2024-01-03

**Authors:** Yiwei Liu, Eleanor A. McQuillen, Pranav S. J. B. Rana, Erin S. Gloag, Matthew R. Parsek, Daniel J. Wozniak

**Affiliations:** ^a^Department of Microbiology, Ohio State University, Columbus, OH 43210; ^b^Department of Microbial Infection and Immunity, Ohio State University College of Medicine, Columbus, OH 43210; ^c^Department of Health and Rehabilitation Sciences, Ohio State University College of Medicine, Columbus, OH 43210; ^d^Department of Biomedical Sciences and Pathobiology, Virginia Maryland College of Veterinary Medicine, Virginia Tech, Blacksburg, VA 24060; ^e^Department of Microbiology, University of Washington School of Medicine, Seattle, WA 98195

**Keywords:** Staphyloxanthin, *Pseudomonas aeruginosa*, *Staphylococcus aureus*, polymicrobial, innate immunity

## Abstract

Bacterial infections are often polymicrobial. *Pseudomonas aeruginosa* and *Staphylococcus aureus* cause chronic co-infections, which are more problematic than mono-species infections. Understanding the mechanisms of their interactions is crucial for treating co-infections. Staphyloxanthin (STX), a yellow pigment synthesized by the *S. aureus crt* operon, promotes *S. aureus* resistance to oxidative stress and neutrophil-mediated killing. We found that STX production by *S. aureus*, either as surface-grown macrocolonies or planktonic cultures, was elevated when exposed to the *P. aeruginosa* exoproduct, 2-heptyl-4-hydroxyquinoline N-oxide (HQNO). This was observed with both mucoid and non-mucoid *P. aeruginosa* strains. The induction phenotype was found in a majority of *P. aeruginosa* and *S. aureus* clinical isolates examined. When subjected to hydrogen peroxide or human neutrophils, *P. aeruginosa* survival was significantly higher when mixed with wild-type (WT) *S. aureus*, compared to *P. aeruginosa* alone or with an *S. aureus crt* mutant deficient in STX production. In a murine wound model, co-infection with WT *S. aureus*, but not the STX-deficient mutant, enhanced *P. aeruginosa* burden and disease compared to mono-infection. In conclusion, we identified a role for *P. aeruginosa* HQNO mediating polymicrobial interactions with *S. aureus* by inducing STX production, which consequently promotes resistance to the innate immune effectors H_2_O_2_ and neutrophils. These results further our understanding of how different bacterial species cooperatively cause co-infections.

## Significance

*P. aeruginosa* and *S. aureus* are two important human pathogens that often cause co-infections. Understanding their polymicrobial interactions is key to treating such infections. HQNO secreted by *P. aeruginosa* is known to antagonize the growth of *S. aureus*. However, we identified a role for sub-lethal levels of HQNO in inducing *S. aureus* STX production. This facilitates a cooperative behavior between the two pathogens, resulting in resistance to host ROS and increased bacterial burden during co-infections in vivo. Our findings contribute to the understanding of complex polymicrobial interactions and suggest a critical role for HQNO in controlling the balance between cooperative and competitive behaviors between *P. aeruginosa* and *S. aureus*.

*Pseudomonas aeruginosa* and *Staphylococcus aureus* are two common microorganisms colonizing cystic fibrosis (CF) airways and chronic wounds (1–4). Co-infection correlates with increased disease severity, compared to mono-infections caused by either species (4–6).

*P. aeruginosa* and *S. aureus* have an intriguingly complicated relationship and have been used as model organisms to investigate polymicrobial interactions. In vitro, *P. aeruginosa* outcompetes *S. aureus*. Many antagonistic mechanisms have been determined in *P. aeruginosa* (7–10). However, of relevance to this study is the *P. aeruginosa* quorum-sensing (QS) system PQS (*Pseudomonas* quinolone signal) which is crucial for antagonizing *S. aureus* (11). The PQS system is involved in the production of 2-heptyl-4-hydroxyquinoline n-oxide (HQNO) which inhibits respiration (12) and promotes small colony variant formation in *S. aureus* (13). This operon is responsible for synthesizing the HQNO precursor HHQ (14), which is converted to HQNO by the PqsL enzyme. In contrast to antagonism in vitro, both pathogens can co-exist in vivo. In fact, interaction with *S. aureus* can benefit *P. aeruginosa* by increased biofilm formation (15), host immune evasion (16), and antibiotic resistance (17, 18). One of the keys to understanding this polymicrobial relationship is the subtle balance between the competitive and cooperative behaviors of these two organisms.

Staphyloxanthin (STX) is a membrane-bound yellow pigment, synthesized by the *crt* operon in *S. aureus* (19–21) and widely produced among clinical and environmental isolates (22). Strains deficient in STX production appear as white colonies on solid media. By functioning as an antioxidant to resist oxidative stress and altering membrane fluidity to combat antimicrobial peptides (APs), STX mediates *S. aureus* resistance to host defense mechanisms (23, 24). Interestingly, a *P. aeruginosa* wound isolate was observed to induce STX production in a co-isolated white variant of *S. aureus* (25). This implies that STX mediates interactions between *P. aeruginosa* and *S. aureus* in vivo.

Given the importance of *P. aeruginosa* and *S. aureus* co-infections and the implication of STX in polymicrobial interactions, here we investigated the role of STX during *P. aeruginosa* and *S. aureus* co-infections. We found that STX production is induced by *P. aeruginosa* HQNO and affords cross-species protection against H_2_O_2_- and neutrophil-mediated killing.

## Results

### *P. aeruginosa* Exoproduct HQNO Induces STX Production in Both Surface- and Planktonic-grown *S. aureus*.

**Fig. 1.**
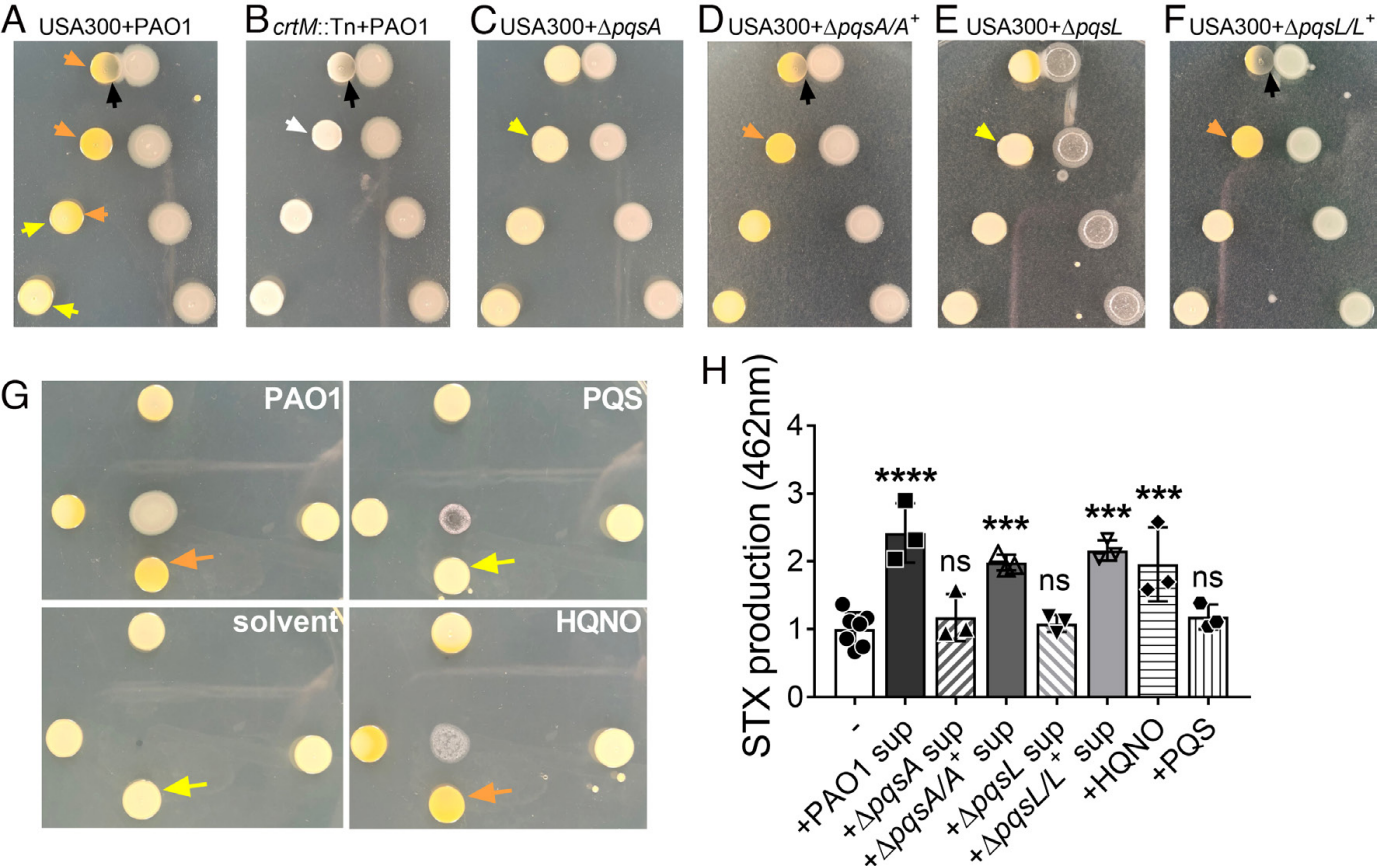
*P. aeruginosa* HQNO induces *S. aureus* STX production. (*A*–*G*) USA300 or *crtM*::Tn was grown at increasing distances from designated *P. aeruginosa* strains, PQS, HQNO, or the solvent in which the molecules were dissolved, on solidified media in a macrocolony proximity assay. Yellow arrows point to USA300 with no pigment change, orange arrows point to USA300 with increased yellow pigmentation, and the white arrow indicates white *crtM*::Tn colonies. The black arrows point to *S. aureus* growth inhibition by *P. aeruginosa*. (*H*) STX production in LB-grown USA300 treated with (+) or without (−) 5% filter-sterilized spent media (sup) of designated *P. aeruginosa* strains, or 5 μM of HQNO or PQS. STX production was quantified by absorbance at OD_462_. The results were normalized to the untreated group. Data are presented as mean ± SD from the results of at least three biological replicates, each with two technical replicates. ***P < 0.001; ****P < 0.0001; ns, not significant, compared to the untreated group, determined by one-way ANOVA.

In our previous study, we examined the contribution of *P. aeruginosa* factors, especially released exopolysaccharide Psl (10), in antagonizing the growth of *S. aureus* in a macrocolony proximity assay. *S. aureus* USA300 was grown at increasing distances from *P. aeruginosa* PAO1 on solidified media (Fig. 1*A*). As expected, the growth of USA300 adjacent to PAO1 was inhibited. Interestingly, we also observed a difference in USA300 pigmentation. USA300 macrocolonies furthest from PAO1 were light yellow, whereas the ones closer to PAO1 were more pigmented, suggestive of elevated STX production. In contrast, macrocolonies of an *S. aureus crtM* transposon mutant (*crtM*::Tn, SAUSA300_2499) deficient in STX production, remained non-pigmented regardless of the distance from PAO1 macrocolonies (Fig. 1*B*). This suggests that an exoproduct released by PAO1 can diffuse through the solidified media to induce STX production in the nearby USA300.

STX production is a stress response of *S. aureus* (26). Since there was increased STX production along with growth inhibition of USA300 when adjacent to PAO1, we investigated whether STX induction by *P. aeruginosa* was a response to general growth inhibition. USA300 macrocolonies were grown at increasing distances from filter disks soaked in ciprofloxacin or daptomycin antibiotics, or PAO1 (*SI Appendix*, Fig. S1). STX induction was only observed for USA300 macrocolonies grown in proximity to PAO1, but not the antibiotics, despite similar levels of growth inhibition. This implies that *S. aureus* STX is induced in response to released *P. aeruginosa* factor(s) and not a general response to growth inhibition.

*P. aeruginosa* can secrete many antagonistic factors, including Psl (10), rhamnolipid (9), HQNO (13), LasA (7), and pyoverdine (8), to inhibit the growth of *S. aureus*. We used the corresponding mutants in PAO1 to determine whether any of these mechanisms were responsible for inducing *S. aureus* STX production. When USA300 and these PAO1 mutants were grown together in the macrocolony proximity assay, Δ*pqsA* was the only mutant that failed to antagonize *S. aureus* and not induce STX production (Fig. 1*C*), while others still antagonized the growth of USA300 and induced STX production (*SI Appendix*, Fig. S2). Chromosomal complementation of Δ*pqsA* (Δ*pqsA/A^+^*) was able to revert the phenotype to the level of PAO1 (Fig. 1*D*). *pqsA* is one of the *P. aeruginosa* PQS biosynthetic operon enzymes responsible for the synthesis of HHQ, the precursor of HQNO (14, 27, 28). To investigate whether either or both products were sufficient for STX induction, USA300 macrocolonies were grown at increasing distances from commercially acquired HQNO and PQS, the solvent used to dissolve the chemicals, or PAO1 (Fig. 1*D*). Increased STX production of USA300 macrocolonies was only observed when grown adjacent to HQNO and PAO1, but not PQS or the solvent. *pqsL* is directly responsible for HQNO synthesis (29). Interestingly, Δ*pqsL* poorly induced STX production in adjacently grown USA300 (Fig. 1*E*). Chromosomal complementation (Δ*pqsL/L^+^*) fully reverted the phenotype comparable to PAO1 (Fig. 1*F*). The above data suggest that the *P. aeruginosa* PQS system, particularly the synthesis of HQNO, is responsible for inducing STX production in surface- grown *S. aureus*.

Since exoproducts are secreted into the spent media during bacterial planktonic growth, we also examined whether *S. aureus* STX could be induced by *P. aeruginosa* exoproducts when grown in planktonic culture. 50% PAO1 spent media demonstrated a strong bactericidal effect on *S. aureus* within 4 h of treatment (10). High concentrations of HQNO (e.g., 400 μM) also inhibit the growth of *S. aureus* (30). To eliminate complications due to potential growth inhibition, USA300 planktonic cultures were grown in LB supplemented with 5% PAO1 spent media, 5 μM of HQNO, or 5 μM of PQS for 16 h. STX was then extracted and quantified as described (19, 20). The presence of PAO1 spent media and HQNO, but not Δ*pqsA* and Δ*pqsL* spent media or PQS, induced *S. aureus* STX production (Fig. 1*H*).

Synthetic CF sputum media (SCFM2) mimics the CF sputum composition and has been used to culture both *P. aeruginosa* and *S. aureus* (31, 32). USA300 was also grown planktonically in SCFM2 supplemented with either PAO1 spent media or HQNO. In both conditions, STX production was significantly higher, compared to USA300 grown in SCFM2 alone, implying that HQNO can induce STX production under in vivo-like conditions (*SI Appendix*, Fig. S3). Overall, the above data indicate that *P. aeruginosa* HQNO is sufficient to induce *S. aureus* STX production, regardless of the mode of growth.

### *P. aeruginosa* Induction of *S. aureus* STX Production Is Prevalent among Clinical Isolates.

STX production varies across *S. aureus* laboratory strains and clinical isolates (33, 34). We screened a collection of 61 *S. aureus* clinical isolates, from CF lung and bloodstream infections (*SI Appendix*, Table S1), and found that the majority of them (78.7%) had increased yellow pigmentation when grown in proximity to PAO1, suggesting STX induction (*SI Appendix*, Fig. S4*A* and Table S2). A similar phenotype was observed with methicillin-sensitive *S. aureus* (MSSA; *SI Appendix*, Fig. S5). *P. aeruginosa* clinical isolates synthesize varying levels of HQNO (35, 36). We also screened 29 *P. aeruginosa* clinical isolates, derived from CF lung and wound infections (*SI Appendix*, Table S1). 72.4% of them induced STX production in USA300, with the representative strains producing HQNO varying from 13.6 μM to 119.6 μM. The remaining isolates did not induce STX production, and the representative strains produced no detectable HQNO (*SI Appendix*, Fig. S4 *B* and *C* and Table S3). Overall, *P. aeruginosa* induction of *S. aureus* STX production was observed in the majority of the examined clinical isolates.

Mucoid conversion, defined by the overproduction of the exopolysaccharide alginate, occurs frequently in *P. aeruginosa* clinical CF strains (37). Mucoid *P. aeruginosa* can co-exist with *S. aureus* better than non-mucoid counterparts, due to reduced production of antagonistic factors, including HQNO (32). Since some of the *P. aeruginosa* clinical isolates tested above were mucoid and induced USA300 STX production (*SI Appendix*, Fig. S4*B*), we examined whether mucoid *P. aeruginosa* could induce STX production in an HQNO-dependent manner. To test this, we used a laboratory *P. aeruginosa* mucoid strain (PAO1 *mucA22*; PDO300) and created a Δ*pqsA* allele in this background. In the macrocolony proximity assay, PDO300 induced STX production in the adjacent USA300 macrocolonies, although without growth inhibition (*SI Appendix*, Fig. S4*D*). However, for the PDO300Δ*pqsA* mutant, STX induction was abolished. USA300 was also grown planktonically in media supplemented with spent media from either PDO300, PDO300Δ*pqsA,* or PAO1Δ*pqsA,* followed by STX extraction and quantification. When grown in the presence of PDO300 spent media, USA300 produced significantly more STX, compared to growth in media alone (*SI Appendix*, Fig. S4*E*). Neither PAO1Δ*pqsA* nor PDO300Δ*pqsA* spent media was able to induce STX production. This suggests that mucoid *P. aeruginosa* also induces *S. aureus* STX production in an HQNO-dependent manner.

### *P. aeruginosa*–Induced STX Production Protects Both *S. aureus* and *P. aeruginosa* from H_2_O_2_-mediated Killing.

**Fig. 2.**
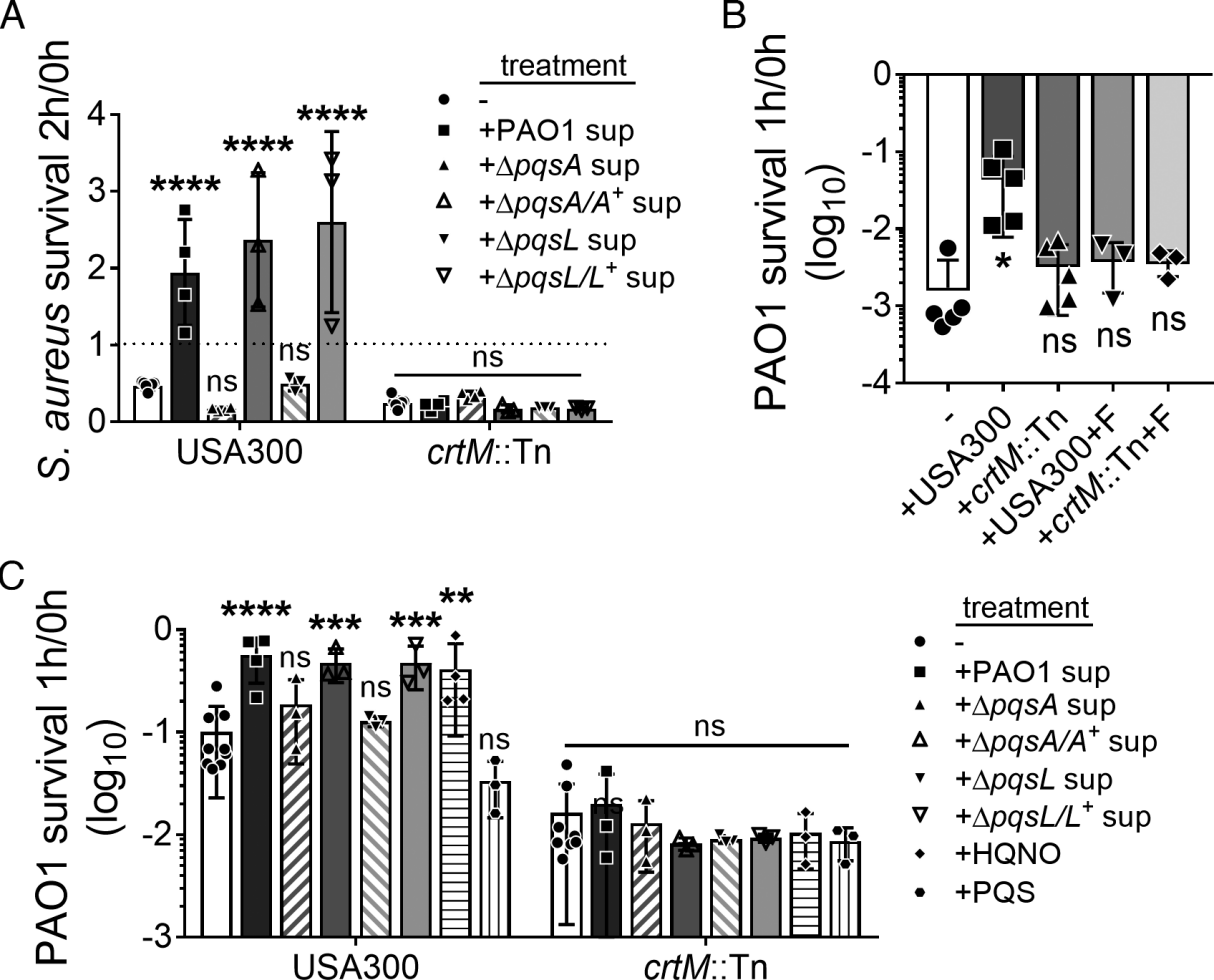
STX induction protects both *S. aureus* and *P. aeruginosa* from H_2_O_2_-mediated killing. (*A*) *S. aureus* USA300 and *crtM::Tn* were pre-treated with or without 5% filter-sterilized spent media (sup) from designated *P. aeruginosa* strains overnight and then subjected to 3% H_2_O_2_-mediated killing for 2 h. The dotted line indicates 100% survival. (*B*) PAO1, alone (−) or mixed with an equal amount of *S. aureus*, was subjected to 3% H_2_O_2_-mediated killing for 1 h. USA300 and *crtM*::Tn were pre-treated with 50 μg/mL flavone (+F) to inhibit STX production and serve as controls. (*C*) PAO1 mixed with an equal amount of *S. aureus* with various treatments was subjected to 3% H_2_O_2_-mediated killing for 1h in LB. USA300 and *crtM*::Tn were pre-treated with or without (−) 5% filter-sterilized *P. aeruginosa* spent media (sup), or 5 μM HQNO or PQS overnight. Bacterial survival is presented as CFUs normalized to the starting CFUs at 0 h. Data are presented as mean ± SD from the results of at least three biological replicates, each with three technical replicates. **P < 0.01; ***P < 0.001; ****P < 0.0001; ns, not significant, compared to the *P. aeruginosa* alone group (*B*) or the no treatment group (*A* and *C*). Statistical differences are determined by two-way ANOVA (*A* and *C*) or one-way ANOVA (*B*).

STX provides *S. aureus* resistance to oxidative stress, as a *crtM* mutant deficient in STX production was more sensitive to H_2_O_2_-mediated killing than WT *S. aureus* (23). However, the survival of *S. aureus* to oxidative stress upon STX induction has not been described. We therefore wanted to determine whether *P. aeruginosa*–induced STX production could be beneficial for *S. aureus* survival in the presence of H_2_O_2_. As previously described, USA300 and *crtM*::Tn were grown overnight in media supplemented with PAO1, Δ*pqsA*, or Δ*pqsL* spent media to induce STX production. The cultures were then subjected to 3% H_2_O_2_–mediated killing for up to 2 h. *S. aureus* survival was quantified by colony-forming units (CFUs) at designated time points, normalized to that of 0h. At 1h of H_2_O_2_ treatment, little difference in survival was observed for USA300 or *crtM*::Tn (*SI Appendix*, Fig. S6*A*). At 2 h, the survival of H_2_O_2_-treated USA300 and *crtM*::Tn, grown without *P. aeruginosa* spent media, reduced to 47% and 25%, respectively (Fig. 2*A*). Prior growth in media supplemented with PAO1, Δ*pqsA/A^+^*, or Δ*pqsL/L^+^* spent media, but not Δ*pqsA* or Δ*pqsL*, significantly increased USA300 survival by >fourfold, compared to USA300 grown in media alone (Fig. 2*A*). There was no significant difference in *crtM*::Tn survival under these conditions. The above data indicate that *P. aeruginosa*–induced STX production can further protect *S. aureus* from H_2_O_2_-mediated killing.

STX is a potent antioxidant and scavenges free radicals via conjugated double bonds (23, 38). We therefore hypothesized that in co-culture, STX may benefit *P. aeruginosa* by protecting it from H_2_O_2_-mediated killing. PAO1 alone, or mixed with an equal amount of *S. aureus*, was treated with 3% H_2_O_2_ for 1 h (Fig. 2*B*). PAO1 survival increased >10-fold when mixed with USA300, compared to PAO1 alone. However, co-culture with *crtM*::Tn did not significantly change PAO1 survival in the presence of H_2_O_2_. We then wanted to confirm that the protection conferred to *P. aeruginosa* from H_2_O_2_-mediated killing by USA300 was due to STX production, rather than other potential functions of *crtM*. To test this, USA300 and *crtM::Tn* were grown overnight in media supplemented with flavone, a plant flavonoid that inhibits *S. aureus* STX production without affecting growth (39). The *S. aureus* cultures were then mixed with PAO1 and treated as above, and bacterial survival quantified (Fig. 2*B*). Flavone completely abolished the protection that USA300 conferred on PAO1 against H_2_O_2_. No difference in the survival of PAO1 was observed when mixed with *crtM*::Tn grown with or without flavone. As expected, with 1 h treatment conditions, survival for both USA300 and *crtM*::Tn was not affected (*SI Appendix*, Fig. S6*B*), suggesting that the difference in PAO1 survival is attributed to STX, rather than the amount of *S. aureus* present. Together, the above data suggest that USA300 protects PAO1 from H_2_O_2_-mediated killing, and this protection is STX-dependent.

Since HQNO induces *S. aureus* STX production, we further tested if *S. aureus* with induced STX production affords better protection to PAO1 from H_2_O_2_. USA300 STX production was induced when grown in LB supplemented with PAO1 spent media or HQNO (Fig. 1*H*). When mixed with the above cultures, the survival of PAO1 challenged with H_2_O_2_ was increased >fivefold, compared to PAO1 mixed with USA300 grown in LB alone (Fig. 2*C*). Supplementing with Δ*pqsA* or Δ*pqsL* spent media or PQS during growth did not increase the ability of USA300 to protect PAO1. This was reversed upon complementation of the deleted genes with corresponding wild-type alleles. No significant difference was found in the survival of PAO1 when mixed with *crtM*::Tn grown with or without any *P. aeruginosa* exoproducts. The survival of USA300 and *crtM*::Tn remained unchanged for all conditions (*SI Appendix*, Fig. S6*C*). The above data indicate that induced STX production in *S. aureus* affords better protection to PAO1 from H_2_O_2_-mediated killing.

In addition to growth in LB, the presence of STX-producing USA300 also protected PAO1 from H_2_O_2_-mediated killing in SCFM2. Moreover, USA300 with increased STX production, induced by PAO1 spent media or HQNO, afforded better protection to PAO1 in SCFM2 compared to USA300 grown in only LB (*SI Appendix,* Figs. S6*D* and S7). The above data suggest that *S. aureus* STX also protects *P. aeruginosa* from H_2_O_2_ killing in environments mimicking the CF airway.

We also examined whether STX can protect mucoid PDO300 from H_2_O_2_ killing (*SI Appendix,* Fig. S8). Compared to PDO300 alone, the presence of USA300 increased the survival of PDO300 by 10-fold. PDO300 survival was further increased when the mixed USA300 had induced STX production due to growth in PDO300 spent media. This was not observed with PDO300Δ*pqsA* or PAO1Δ*pqsA* spent media. The above data indicate that STX protection from H_2_O_2_-mediated killing extends to *P. aeruginosa* mucoid strains.

### STX Protects *P. aeruginosa* from Killing by Human Neutrophils.

**Fig. 3.**
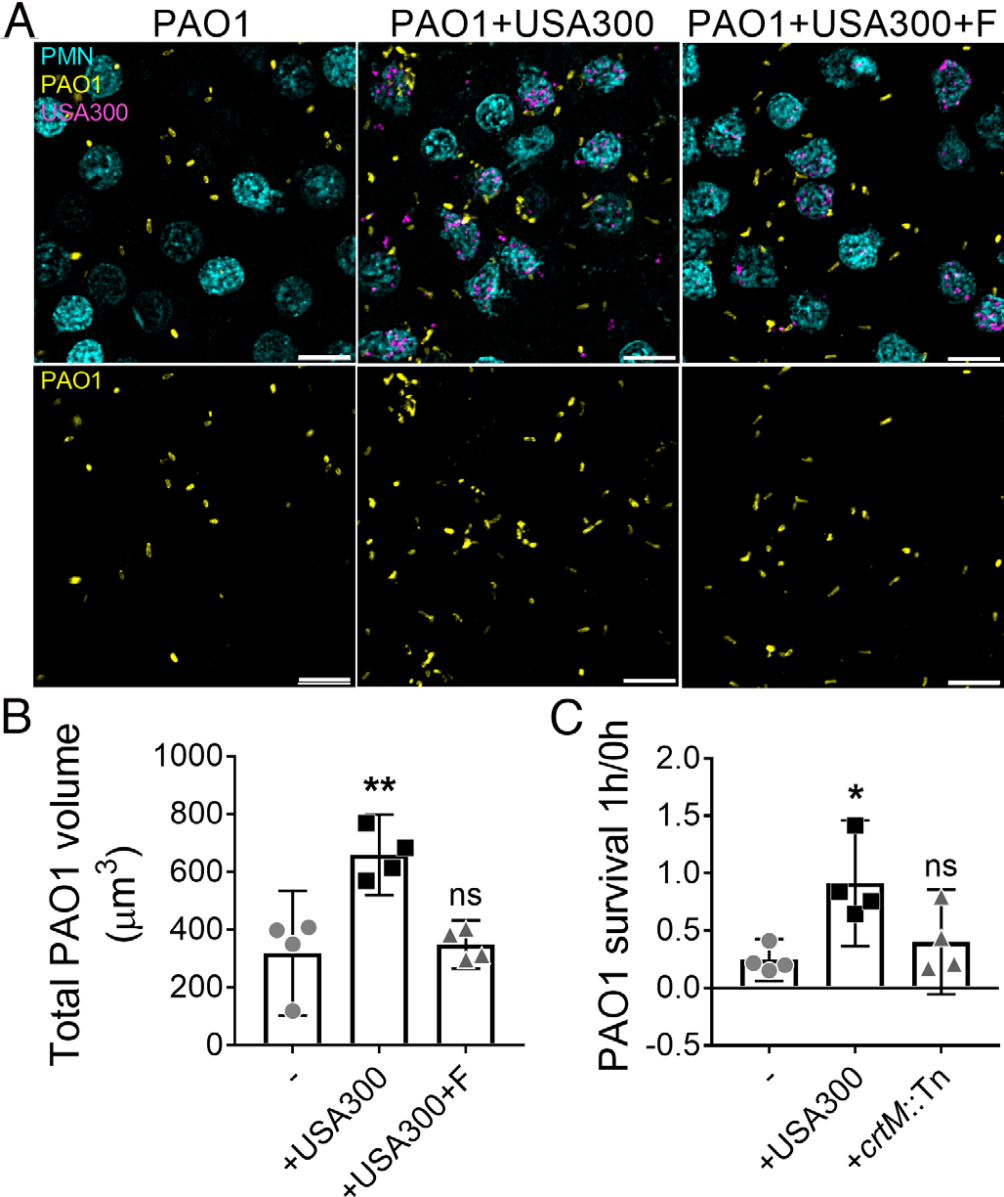
STX can protect *P. aeruginosa* from killing by human neutrophils. (*A* and *B*) PAO1-TdTomato, either alone or mixed with an equal amount of USA300-GFP, was subjected to adhered human neutrophil (PMN) for 1 h to assess PAO1 survival (MOI = 10 for each species). USA300-GFP was pre-treated with 50 μg/mL flavone (+F) to inhibit STX production. (*A*) Representative images of PAO1 and USA300 infected neutrophils. (Scale bar: 40 μm.) (*B*) Total PAO1 volume was quantified by measuring fluorescence intensity. Data are presented as mean ± 95%CI from the results of four biological replicates, each with six technical replicates. (*C*) PAO1, either alone or mixed with an equal amount of USA300 or *crtM*::Tn, was subjected to human neutrophil killing for 1 h (MOI = 10 for each species). PAO1 survival is presented as CFUs normalized to the starting CFUs at 0 h. Data are presented as mean ± 95%CI from the results of four biological replicates, each with three technical replicates. *P < 0.05; **P < 0.01; ns, not significant, compared to PAO1 mono-infection determined by one-way ANOVA. *S. aureus* survival is quantified in *SI Appendix*, Fig. S9.

Neutrophils can combat pathogens by generating both intra- and extra-cellular reactive oxygen species (ROS) (40). STX mediates *S. aureus* resistance to neutrophil killing by serving as an antioxidant (23, 38). In the experiments outlined above, we demonstrated that STX protected *P. aeruginosa* from oxidative stress generated by H_2_O_2_ (Fig. 2 *B* and *C*). We wanted to determine if *S. aureus* STX could also protect *P. aeruginosa* from killing by human neutrophils. To test this, human peripheral blood–derived neutrophils were infected with fluorescently tagged USA300 and PAO1. To inhibit STX production, USA300 was grown in the presence of flavone as described (Fig. 2*B*). After 1 h incubation, the infection was visualized by wide-field fluorescent microscopy (Fig. 3*A*). Imaging analysis was performed to quantify the volume of PAO1 fluorescent signal, as an indication of PAO1 survival. This revealed that PAO1 survival was higher in the co-infection with USA300, compared to PAO1 mono-infection (Fig. 3*B*). PAO1 survival in the presence of flavone-treated USA300 was equivalent to that of PAO1 mono-infection (Fig. 3*B*). To support these observations, human peripheral blood–derived neutrophils were infected with PAO1 alone, or with USA300 or *crtM*::Tn for 1 h, and bacterial survival quantified by CFU (Fig. 3*C*). A significant increase was found in the survival of PAO1 when co-infected with USA300, but not with *crtM*::Tn, compared to PAO1 alone. *S. aureus* survival remained unchanged in mono-infections or co-infections with PAO1 (*SI Appendix,* Fig. S9). The above data suggest that *S. aureus* STX protects *P. aeruginosa* from killing by human neutrophils.

### STX Enhances *P. aeruginosa* Infection In Vivo.

**Fig. 4.**
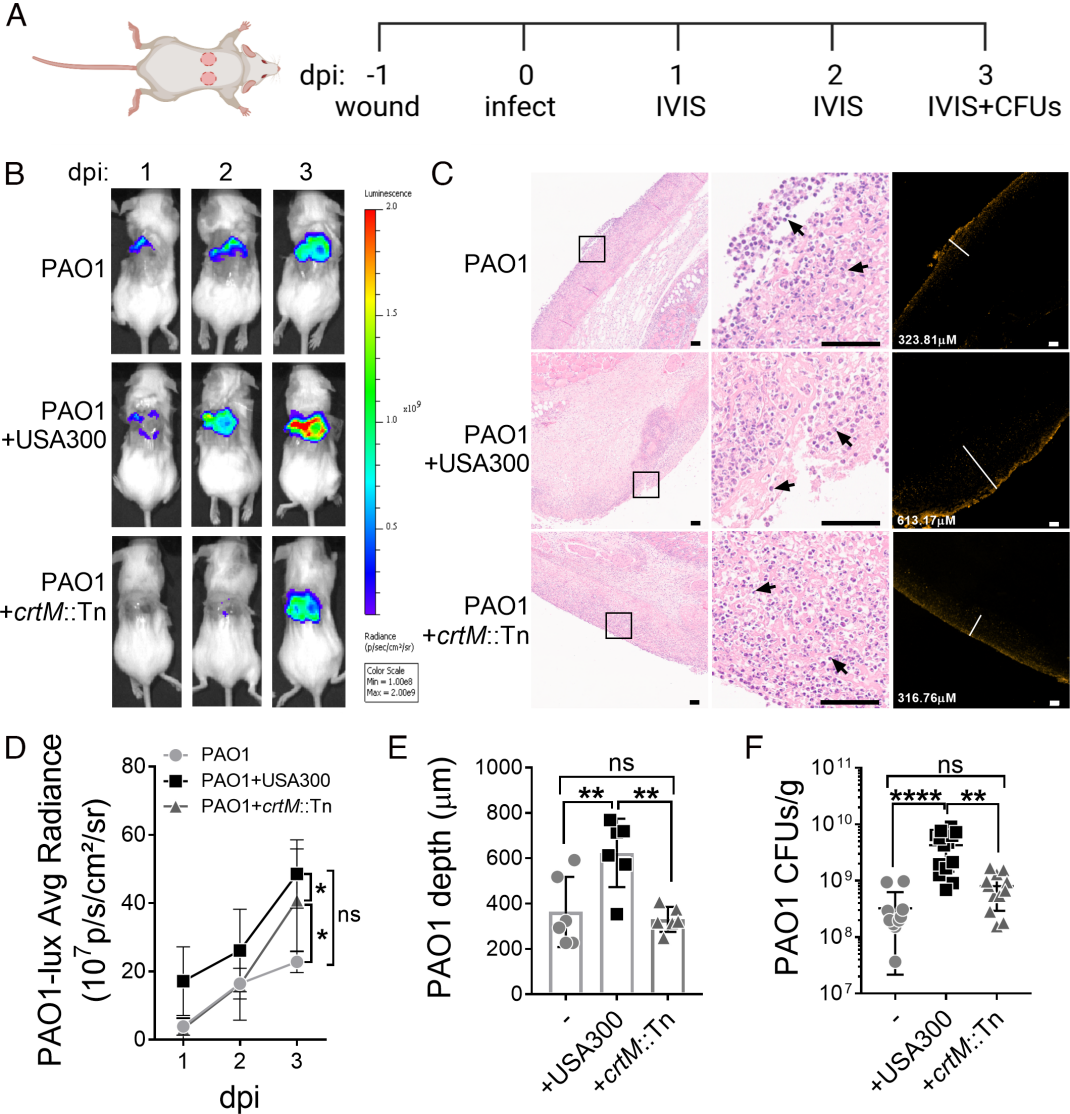
STX promotes the establishment of *P. aeruginosa* infection in vivo. (*A*) Schematic of the murine wound model and course of infection. Two identical full-thickness dorsal wounds were generated using 6 mm punch biopsies. After 24 h, the wounds were mono-infected with luminescent PAO1, USA300, or *crtM*::Tn, or co-infected with PAO1 and USA300 or *crtM*::Tn. On 1, 2, and 3 days post-infection (dpi), IVIS was used to monitor PAO1 burden among all groups. Wounds were harvested and homogenized to plate for both PAO1 and *S. aureus* CFUs on 3 dpi. (*B*) Representative images of luminescent PAO1 detected using IVIS on murine wounds throughout the 3 d of infection. (*C*) Representative images of H&E (*Left* and *Middle* panels) and IF (*Right* panel) stained adjacent wound sections (4 μm). The *Left* panel shows the wound beds in low magnifications. Magnified boxed areas are shown in the *Middle* panel, with black arrows pointing to neutrophil infiltration. The *Right* panel shows the presence of immunofluorescently labeled PAO1 and the white lines measure the depth of PAO1 penetration (labeled in the bottom left corner). (Scale bar: 100 μm.) (*D*) Luminescent signal intensity of PAO1*-lux* was quantified by the average radiance. Significant differences were determined by comparing the area under the curve (*SI Appendix*, Fig. S10*A*). Data presented as mean ± 95%CI from the results of >12 biological replicates. (*E*) The depth of PAO1 penetration into the wound with PAO1 mono-infection or co-infection with USA300 or *crtM*::Tn. Data are presented as mean ± 95%CI from the results of six biological replicates. (*F*) PAO1 CFUs per gram of wound tissue (CFUs/g) among all groups was quantified. Data are presented as mean ± 95%CI from the results of >12 biological replicates with three technical replicates. *P < 0.05; **P < 0.01; ****P < 0.0001; ns, not significant, determined by one-way ANOVA (*D* and *E*) or the Kruskal–Wallis test (*F*). *S. aureus* survival is quantified in *SI Appendix*, Fig. S10.

Our findings so far have demonstrated that STX protects *P. aeruginosa* from killing by H_2_O_2_ and neutrophils (Figs. 2 and 3). Since neutrophils are one of the first innate immune cells recruited to the infection site (41), we hypothesized that STX could also protect PAO1 during the early stages of infection. To test this, a dermal full-thickness murine wound model was used to examine *P. aeruginosa* and *S. aureus* co-infection (42). Briefly, two dorsal wounds were generated by punch biopsies and infected with either a luminescent tagged PAO1 strain, USA300, *crtM*::Tn, or both species. The infection was allowed to progress for 3 d to focus on early bacterial infection and innate immunity (Fig. 4*A*). By using an in vivo imaging system (IVIS), we monitored the burden of the bioluminescent PAO1 daily by measuring signal intensity. Throughout the 3-d infection period, PAO1 burden was significantly higher in mice co-infected with USA300, than those only infected with PAO1 or co-infected with *crtM*::Tn (Fig. 4 *B* and *D* and *SI Appendix,* Fig. S10*A*). No significant difference was found between PAO1 mono-infection and *crtM*::Tn co-infection. The above data suggest that *S. aureus* STX increases the PAO1 burden throughout infection.

On day 3 post infection, wound tissues were harvested and processed for either hematoxylin and eosin (H&E) staining or immunofluorescence (IF) labeling of PAO1, to assess inflammation and PAO1 localization respectively. We observed infiltration of mixed inflammatory cells, which predominantly consisted of neutrophils, into the wound bed for all groups (Fig. 4 *C*, *Left* and *Middle* panels). IF staining showed that PAO1, when co-infected with USA300, penetrated significantly deeper into the tissue (623.8 ± 150.9 μm) compared to mono-infection (363.5 ± 154.8 μm) or co-infection with *crtM*::Tn (330.2 ± 54.81 μm) (Fig. 4 *C* and *E*). Total PAO1 fluorescent signal from immunofluorescent images was quantified as another indication of PAO1 burden and was higher when PAO1 was co-infected with USA300, but not *crtM*::Tn or PAO1 alone (*SI Appendix,* Fig. S10*B*). The above results were corroborated by enumerating bacteria by CFU, after 3 d of infection (Fig. 4*F*). When PAO1 was co-infected with USA300, but not *crtM*::Tn, there was a significant 10-fold increase in PAO1 burden, compared to PAO1 mono-infection. There was no significant difference in the bacterial burden between USA300 or *crtM*::Tn (*SI Appendix*, Fig. S10*D*). Overall, the above data indicate that STX-producing *S. aureus* promotes *P. aeruginosa* wound colonization during the early stages of infection, demonstrating a clear role for STX in enhancing *P. aeruginosa* infection in vivo.

## Discussion

In this study, we identified a role for *P. aeruginosa* HQNO in inducing *S. aureus* STX production (Fig. 1). The well-studied influence of HQNO on *S. aureus* is that, at high concentrations (400 μM), it antagonizes growth by inhibiting respiration (30, 43, 44), and promotes the formation of *S. aureus* small colonies, slow-growing, and non-pigmented variants (13, 30, 44, 45). However, we observed increased STX production in the presence of sub-lethal HQNO concentrations (5 μM). It is likely that varying concentrations of HQNO may elicit different responses in *S. aureus*. Many factors contribute to the local concentration of HQNO. The spatial distribution of bacteria can alter local QS signal concentration (46). In Fig. 1*A*, USA300 growth was inhibited by the adjacent PAO1 with high local HQNO concentration. The antagonism disappeared and STX induction was observed as the HQNO concentration decreased, due to the increased distance between the two bacteria macrocolonies. As the distance between the two colonies further increased, HQNO concentration was likely too low to induce STX production. In addition, different *P. aeruginosa* laboratory and clinical strains can produce varying amounts of HQNO, ranging from 0 to 50 μM (35, 36). Mucoid *P. aeruginosa* can co-exist with *S. aureus* better than the non-mucoid counterparts (32). Exogenous alginate can mitigate the killing of *S. aureus* by down-regulating HQNO production and other antagonistic factors in both mucoid and non-mucoid strains (47). Consistent with this, we observed reduced USA300 antagonism and induction of STX in some mucoid clinical isolates and PDO300 in an HQNO-dependent manner (*SI Appendix,* Fig. S4 *B*–*E*). The above evidence suggests that mucoid *P. aeruginosa* can create a low-HQNO environment that promotes *S. aureus* niche compatibility. This allows for the induction of STX which is beneficial for both species. In addition to mucoid strains, some non-mucoid *P. aeruginosa* clinical isolates can also induce STX production without inhibiting the growth of adjacent USA300 (*SI Appendix,* Fig. S4*B*), possibly due to reduced HQNO production in these isolates. Indeed, the clinical isolates that we examined produced varying levels of HQNO, which positively correlated with their ability to induce STX (*SI Appendix,* Fig. S4 *B* and *C*).

Apart from spatial distribution and strain variations, nutrient availability and host factors may also alter HQNO concentration (46, 48, 49). Overall, we predict that different concentrations of HQNO may be responsible for controlling the balance between cooperative and competitive behaviors among *P. aeruginosa* and *S. aureus*. Interestingly, Ibberson et al. recently discovered that HQNO can mediate the spatial structure of *P. aeruginosa* and *S. aureus* in wound infections (50). Collectively, both of our findings support the critical role of HQNO in modulating *P. aeruginosa* and *S. aureus* interactions during co-infections.

The mechanism(s) of how HQNO induces STX remains unclear. It seems to correlate with, but is not dependent on growth inhibition, as most of the STX-inducing *P. aeruginosa* clinical isolates can inhibit *S. aureus* growth (*SI Appendix,* Fig. S4*B*). This implies that STX may be induced in response to HQNO-mediated antagonism. HQNO can interfere with the electron transfer system in bacteria and mitochondria, which results in the production of ROS (12, 51–53). It is possible that *S. aureus* produces more STX to counteract HQNO-generated ROS. However, results from our experiments suggest otherwise. PAO1-mediated growth inhibition of USA300 and *crtM*::Tn were comparable in both surface-grown colonies (Fig. 1*A*) and planktonic co-cultures (*SI Appendix,* Fig. S11). Moreover, no STX induction was found in USA300 when treated with a sublethal concentration of H_2_O_2_, or ciprofloxacin which results in ROS production (54) (*SI Appendix,* Figs. S1*A* and S12). In addition, we examined the specificity of HQNO-induced STX production. *Burkholderia cenocepacia* produces 4-hydroxy-3-methyl-2-alkyquinolines which are structurally similar to HQNO (55). Neither of the two *B. cenocepacia* strains that we tested inhibited the growth of USA300, nor significantly induced STX production (*SI Appendix,* Fig. S13). One of them, however, demonstrated a modest induction ability, though much lower than PAO1 (*SI Appendix,* Fig. S13 *A* and *B*, c2), warranting future investigations into the potential cooperative behaviors between *S. aureus* and *B, cenocepacia*. Overall, we speculate that the induction of STX by HQNO may be a result of *S. aureus* specifically sensing *P. aeruginosa*–derived HQNO signals. Interspecies signaling is a key factor contributing to polymicrobial interaction and spatial distribution. There is evidence that *S. aureus* may have membrane receptors for another *P. aeruginosa* QS molecule, acyl homoserine lactone (56, 57), but little is known about HQNO. Interestingly, STX is produced by some *S. aureus* clinical isolates but is not induced by the adjacent PAO1 (*SI Appendix,* Fig. S4*A*). This suggests that these isolates may be blind to sensing HQNO. By examining the clinical isolates’ genomes and using STX induction as an output to screen for HQNO-unresponsive variants, future studies will uncover the mechanism(s) of how *S. aureus* senses *P. aeruginosa* HQNO and elevates STX production.

One of the key findings of this study was that STX afforded cross-species protection to *P. aeruginosa* from H_2_O_2_- and neutrophil-mediated killing. Both *S. aureus* and *P. aeruginosa* can colonize the same niche in vivo, within several μm of each other (2, 50). Since the membrane-bound STX has the capability to scavenge free radicals (38), we hypothesize that this creates a low-ROS sink around *S. aureus* cells. This unique microenvironment may, in turn, confer benefits to neighboring *P. aeruginosa* (*SI Appendix,* Fig. S14). In accord with this, STX not only protected *S. aureus* but also *P. aeruginosa* from H_2_O_2_- and neutrophil-mediated killing (Figs. 2 and 3). In addition, co-infection of murine wounds with STX-producing USA300 promoted increased PAO1 burden than the STX-deficient *crtM*::Tn (Fig. 4), despite little difference observed in pathology (*SI Appendix,* Fig. S10*C*). We speculate that the in vivo fitness afforded to PAO1, by USA300, is due to the antioxidant nature of STX. However, we acknowledge that other host innate immune effectors, excluding neutrophil and H_2_O_2_, may play a role in STX-mediated protection. Furthermore, we cannot exclude the possibility that additional STX-independent factor(s) produced by USA300 may enhance PAO1 infection.

Apart from PAO1, we also quantified *S. aureus* survival. In accord with the findings by Liu et al., *crtM*::Tn survival, compared to USA300, was modestly lower when subjected to H_2_O_2_ killing for 2 h (Fig. 2*A*) and in the murine wound infection (*SI Appendix,* Fig. S10*D*). Since HQNO can induce STX production (Figs. 1 and 2), we also examined whether the presence of PAO1 was beneficial for USA300 in vivo. The survival of USA300 and *crtM*::Tn was reduced during co-infection with PAO1, compared to mono-infections (*SI Appendix,* Fig. S10*D*). However, the *S. aureus* burden remained high (10^7^ ~ 10^8^ CFU/g). This is consistent with previous findings that the in vivo wound environment can promote co-existence, despite an antagonistic relationship (3, 49, 50, 58). Interestingly, the ratio of *crtM*::Tn to that of USA300, during co-infection with PAO1, was lower compared to *S. aureus* mono-infections (*SI Appendix,* Fig. S10*E*). Given that *crtM*::Tn was not more sensitive to antagonism by PAO1 than USA300 (*SI Appendix,* Fig. S11), we speculate that this difference may be attributed to the relatively higher survival of USA300 than *crtM*::Tn when co-infected with PAO1. The presence of PAO1 may induce STX production in USA300 which promotes resistance to host ROS and better survival, but not in *crtM*::Tn. Unfortunately, we were unable to extract and directly quantify STX levels from the homogenized wounds, due to contamination by host debris.

In summary, we identified a role for *P. aeruginosa* HQNO in inducing *S. aureus* STX production which is prevalent among clinical isolates. We also identified a cooperative behavior between the two pathogens during co-infection, resulting in resistance to H_2_O_2_- and neutrophil-mediated killing and increased *P. aeruginosa* burden in vivo (*SI Appendix,* Fig. S14). Overall, our findings add another layer to the already complex interaction between *P. aeruginosa* and *S. aureus* in vivo and highlight the need for future investigation in treating polymicrobial infections.

## Materials and Methods

Full and detailed materials and methods can be found in *SI Appendix*.

### Bacterial Strains and Growth Conditions.

All bacterial strains and plasmids are listed in *SI Appendix*, Table S1. Gene deletion constructs were incorporated into the *P. aeruginosa* genome using homologous recombination (59). Chromosomal complementation of gene deletions in *P. aeruginosa* was performed as previously described (60). The presence of transposon insertion for *S. aureus crtM*::Tn was verified by PCR (61). All planktonic cultures were grown at 37 °C with 200-rpm shaking for 16 h. *S. aureus* planktonic culture was grown in either lysogeny broth (10 g/L tryptone, 5 g/L yeast extract, 10 g/L NaCl; LB) or SCFM2 (62). *P. aeruginosa* planktonic culture was grown in either LB with no salt (LBNS) or SCFM2. For macrocolony proximity assay, *P. aeruginosa* and *S. aureus* were grown on lysogeny agar (LB supplemented with 1.5% agar; LA).

### Macrocolony Proximity Assay.

Macrocolonies were grown by inoculating 5 µL of overnight bacterial culture onto the surface of LA in two sets of experiments. First, *P. aeruginosa* or *B. cenocepacia* and *S. aureus* were spotted on opposite sides of the plate with distances of 0 cm, 1 cm, 2 cm, and 3 cm in between. Second, 5 µL of either PAO1 culture, 50 µM HQNO or PQS, the solvent (methanol and ethanol), or antibiotics (10 mg/mL daptomycin or 1 mg/mL ciprofloxacin) was spotted onto the center of the plate, and the USA300 macrocolonies were spotted 0.5 cm, 1 cm, 2 cm and 3 cm away from the center. The plate was incubated at 37 °C overnight, and the *S. aureus* colonies were examined for their survival and pigment production.

### STX Production in Planktonic Culture.

*P. aeruginosa* overnight cultures were normalized to OD_600_ 2.5, and filter sterilized to collect cell-free spent media. To induce STX production, early-stationary phase *S. aureus* cultures (OD_600_ = 1) were supplemented with either 5% or 20% (v/v) *P. aeruginosa* spent media, 5 µM of PQS or HQNO (Cayman Chemicals) or sublethal concentrations of H_2_O_2_. For PAO1 and its variants, 5% spent media (v/v) was added to *S. aureus.* Since PDO300 produces less HQNO (32), 20% spent media (v/v) of PDO300 and its variants was added to *S. aureus.* To inhibit STX production, *S. aureus* was grown in LB supplemented with 50 μg/mL flavone (Sigma-Aldrich) overnight.

### STX Extraction.

Extraction and quantification of STX were carried out as previously described with modifications (19). In brief, overnight cultures of *S. aureus* were normalized to an OD_600_ of 3. After centrifugation, the pellet was resuspended in 250 µL of methanol and incubated at 55 °C for 3 min. The samples were centrifuged to collect the supernatant and measured for absorbance at OD_462_ using a plate reader (SpectraMax® i3x; Molecular Device).

### H_2_O_2_-Mediated Killing Assay.

This assay was carried out as previously described (23) with modifications. Overnight cultures of *P. aeruginosa* and *S. aureus* were diluted to OD_600_ 0.5 in fresh LB. They were either combined at a 1:1 ratio or separately subjected to 3% H_2_O_2_ (Spectrum Chemical) and incubated at 37 °C with 200 rpm shaking for up to 2 h. Aliquots were taken at every hour, treated with 2,000 U/mL catalase (Sigma-Aldrich), serially diluted, and plated on Difco™ *Pseudomonas* Isolation Agar (PIA) and BBL™ Mannitol Salt Agar (MSA) to enumerate for CFUs of *P. aeruginosa* and *S. aureus*, respectively. Bacterial survival at each time point was normalized to the CFUs at 0 h.

### Neutrophil Isolation.

Informed written consent was obtained from all four healthy donors before the collection of peripheral blood for isolating primary human neutrophils. All procedures were approved by the Ohio State University Institutional Review Board (IRB-2009H0314). Neutrophils were isolated as previously described (63).

### Neutrophil Killing Assay.

This assay was carried out as previously described with modifications (64). *P. aeruginosa* and *S. aureus* overnight cultures were normalized to an OD_600_ of 0.5 and opsonized with 20% human serum (CompTech) for 30 min at 37 °C. The two bacteria were then either mixed at a 1:1 ratio or separately incubated with neutrophils statically for 1 h at 37 °C (MOI = 10 for each bacterial species). The samples were centrifuged at 18,000 × *g* for 10 min to lyse the neutrophils and release internalized bacteria. The pellets were resuspended in HBSS, serially diluted, and plated on PIA and MSA to enumerate CFUs. Bacterial survival was normalized to the CFUs at 0 h.

For microscopy analysis, neutrophils were seeded on poly-l-lysine coated coverslips in HBSS supplemented with 100 μM CellTracker™ Blue (Invitrogen) for 30 min at 37 °C, 5% CO_2_. USA300 was grown overnight in LB supplemented with or without 50 μg/mL flavone. Attached neutrophils were infected with fluorescently tagged PAO1, USA300, or both species for 1 h at 37 °C, 5% CO_2_ (MOI = 10 for each bacterial species). Unattached cells were washed away with HBSS. Coverslips were fixed in 4% paraformaldehyde for 30 min at room temperature, mounted to slides using Prolong™ Gold antifade reagent (Invitrogen), and visualized using a Nikon Ti2 wide field microscope fitted with a 60× oil objective. Total volume of bacteria was quantified as described in *SI Appendix*.

### Dermal Full-Thickness Murine Wound Infection.

This assay was carried out as previously described with modifications (42). Six-week-old female BALB/c mice were used in this experiment. For each mouse, two identical full-thickness dorsal wounds were generated with a 6-mm punch biopsy tool (Integra™ Miltex®) and bandaged with a Tegaderm dressing (3M). After 24 h, each wound was infected with mid-log bacterial cultures containing 5 × 10^6^ cells of either PAO1 containing a constitutively expressed luminescent marker (65), USA300 or *crtM*::Tn, or both species. A total of seven animals were used for each group. To assess PAO1 burden throughout infection, the wound luminescence was imaged daily with an IVIS Lumina II optical imaging system (PerkinElmer Inc.). The average radiance of PAO1-lux on each animal was used to access the PAO1 burden throughout infection. Three days post infection, the wounded tissues were collected, homogenized, serially diluted, plated on PIA and MSA, and incubated at 37 °C overnight. CFUs were calculated per gram of tissue.

### H&E and IF Staining and Pathology Analysis on the Wound Tissues.

Three days post infection, wounds were harvested, fixed in 4% paraformaldehyde for a week, transferred into 100% ethanol, and sent to HistoWiz. The tissues were embedded in paraffin, sectioned longitudinally (4 μm), and stained with H&E. As for the IF staining, the slides were deparaffinized, blocked with 3% bovine serum albumin, incubated with primary *P. aeruginosa* antibody (66) (1:500 dilution) and secondary antibody (Alexa Fluor^TM^ 647 chicken anti-rabbit IgG, Invitrogen; 1:500 dilution). They were visualized by microscopy (Nikon ECLIPSE Ti2) using a 4× objective. Six wounds were imaged for each group. The depth of PAO1 penetration into the wound and total pixel count were measured by NIS-elements AR software.

### Statistical Analysis.

Statistical significance was determined using either ANOVA or the Kruskal–Wallis test after the Shapiro–Wilk test for normality. Analyses were performed using GraphPad Prism v.7 (GraphPad Software). Statistical significance was determined using a *P*-value < 0.05.

## Supplementary Material

Appendix 01 (PDF)

## Data Availability

All study data are included in the article and/or *SI Appendix*.
