# Supplementary material for: A bacterial pigment provides cross-species protection from H2O2- and neutrophil-mediated killing
Source: Proc Natl Acad Sci U S A. 2024 Jan 3;121(2):e2312334121. doi: 10.1073/pnas.2312334121 (PMC10786307; doi:10.1073/pnas.2312334121)
Supplement: Supplementary file 1 — Appendix 01 (PDF) [file pnas.2312334121.sapp.pdf]

Supporting Information for

A BACTERIAL PIGMENT PROVIDES CROSS-SPECIES PROTECTION FROM H_2O_2- AND NEUTROPHIL-MEDIATED KILLING

Yiwei Liu[1,2], Eleanor A. McQuillen[3], Pranav S. J. B. Rana[1,2], Erin. S. Gloag[4], Matthew R. Parsek[5], and Daniel J. Wozniak[1,2] *

[1] Department of Microbiology, Ohio State University, Columbus, OH 43210

[2] Department of Microbial Infection and Immunity, Ohio State University College of Medicine, Columbus, OH 43210

[3] Department of Health and Rehabilitation Sciences, Ohio State University College of Medicine, Columbus, OH 43210

[4] Department of Biomedical Sciences and Pathobiology, Virginia Maryland College of Veterinary Medicine, Virginia Tech, Blacksburg, VA 24060

[5] Department of Microbiology, University of Washington School of Medicine, Seattle, WA 98195

* Corresponding author: Daniel J. Wozniak

Email: Daniel.Wozniak@osumc.edu

This PDF file includes:

Supplementary text

Figures S1 to S13

Tables S1 to S3

SI References

Supplementary materials and methods:

HQNO quantification

HQNO production in the representative *P. aeruginosa* clinical isolates was performed as previously described (1). Briefly, bacterial overnight cultures grown in LB were centrifuged to obtain cell-free spent media. Each sample was diluted with an equivalent volume of methanol containing the internal standard (20 mg/L of d7-quinoline). The solution was then centrifuged at 13,000g for 15 min to obtain supernatant. HQNO concentration was then measured by Liquid Chromatograph Mass Spectrometer.

Neutrophil isolation

Informed written consent was obtained from all 4 healthy donors before the collection of peripheral blood for isolating primary human neutrophils. All procedures were approved by the Ohio State University Institutional Review Board (IRB-2009H0314). Neutrophils were isolated as previously described(2). Briefly, heparinized blood from healthy human donors was collected in saline. Ficoll-Paque® PLUS (GR Healthcare) was layered on top of the blood and then centrifuged at 404 × *g* for 40min at 23˚C. The pellet was then resuspended in an equal volume of 3% cold Dextran in 0.9% NaCl and allowed for sediment for 20min on ice. The upper layer was centrifuged at 665 × *g* for 10min at 4˚C. The resulting pellet was resuspended in cold endotoxin-free H_2O for 30s to lyse red blood cells before 1.8% NaCl solution was immediately added to restore isotonicity. The sample was centrifuged at 131 × *g* for 3min at 4˚C, and the pellet containing neutrophils was resuspended in HBSS (without calcium, magnesium, or phenol red; Corning) and counted in a hemocytometer chamber(3).

Neutrophil killing assay

This assay was carried out as previously described with modifications (4). *P. aeruginosa* and *S. aureus* overnight cultures were normalized to an OD_{600} of 0.5, washed with HBSS, and opsonized

with 20% human serum (CompTech) for 30min at 37˚C. The two bacteria were then either mixed at a 1 : 1 ratio or separately incubated with neutrophils statically for 1h at 37˚C (MOI = 10 for each bacterial species). The samples were centrifuged at 18000 x *g* for 10min to lyse the neutrophils and release internalized bacteria. The pellets were resuspended in HBSS, serially diluted, and plated on PIA and MSA to enumerate CFUs. Bacterial survival was normalized to the CFUs at 0h.

For microscopy analysis, neutrophils (2 x 10^6 cells per well) were seeded on poly-l-lysine coated coverslips in HBSS supplemented with 100 μM CellTracker™ Blue (Invitrogen) for 30min at 37˚C, 5% CO_2. Fluorescently tagged *P. aeruginosa* (PAO1-TdTomato)(5) was grown overnight in LB supplemented with 300ug/mL of carbapenem. Fluorescently tagged *S. aureus* (USA300-GFP)(6) was grown overnight in LB supplemented with or without 50μg/mL flavone. Attached neutrophils were infected with PAO1-TdTomato, USA300-GFP, or both species for 1h at 37˚C, 5% CO_2 (MOI = 10 for each bacterial species). Unattached cells were washed away with HBSS. Coverslips were fixed in 4% paraformaldehyde for 30min at room temperature, mounted to slides using Prolong™ Gold antifade reagent (Invitrogen), and visualized using a Nikon Ti2 wide field microscope fitted with a 60x oil objective. 6 images with Z-stacks of 0.3μm step size were taken for each sample for each replicate. Using the NIS-elements AR software, images were clarified, deconvoluted, and thresholded to quantify the total volume of bacteria. Representative images shown in Figure 6C were presented as a maximum intensity projection, created from the original 3D images by the software.

Dermal full-thickness murine wound infection

This assay was carried out as previously described(7) with modifications. All procedures were approved by the Ohio State University Institutional Animal Care and Use Committee (2017A00000028-R1; 2008R0135-R1; 2011R00000021-R1). Briefly, 6-week-old female BALB/c mice were anesthetized using isoflurane gas, and the dorsal area was shaved. The dorsal area was then sterilized with ethanol and two identical full-thickness dorsal wounds were generated

with a 6mm punch biopsy tool (Integra™ Miltex®) and bandaged with a Tegaderm dressing (3M). The mice were allowed to recover for 24h before infection. For infection, mid-log *P. aeruginosa* and *S. aureus* (OD_{600} = 0.5) were washed and resuspended in 0.9% endotoxin-free saline. Each wound was infected with bacterial cultures containing 5x10^6 cells of either PAO1 (containing a constitutively expressed luminescent marker(8)), USA300 or *crtM*::Tn, or both species. A total of 7 animals were used for each group. To assess PAO1 burden throughout infection, mice were anesthetized, and the wound luminescence was imaged daily with an IVIS Lumina II optical imaging system (PerkinElmer Inc.). The acquired images were scaled to the radiance of 1e8 to 2e9. The average radiance of PAO1-lux on each animal was used to access the PAO1 burden throughout infection. Three days post infection, mice were euthanized by CO_2 inhalation. The wounded tissue was collected, weighed, and placed in separate tubes containing 1 mL of PBS. All samples were homogenized with a Pro Scientific Bio-Gen Series Pro200 hand-held homogenizer for 45 s. The resulting solutions were serially diluted, plated on PIA and MSA, and incubated at 37 °C overnight. CFUs were calculated per gram of tissue.

H&E and IF staining, and pathology analysis on the wound tissues

3 days post infection, wounds were harvested, fixed in 4% paraformaldehyde for a week, transferred into 100% ethanol, and sent to HistoWiz. The tissues were embedded in paraffin, sectioned longitudinally (4µm), and stained with H&E. Digital skin sections were subjectively assessed by HistoWiz for the severity and extent of inflammation to provide pathology scoring. As for the IF staining, the slides were deparaffinized and blocked with 3% bovine serum albumin supplemented with 50 mM glycine, 0.05 % Tween20, and 0.1% Triton X-100 at 4˚C overnight. Slides were then incubated with primary *P. aeruginosa* antibody(9) (1 : 500 dilution) at 4˚C overnight and secondary antibody (Alexa Fluor™ 647 chicken anti-rabbit IgG, Invitrogen; 1 : 500 dilution) at room temperature for 1h. They were visualized by microscopy (Nikon ECLIPSE Ti2)

using a 4x objective. 6 wounds were imaged for each group. The depth of PAO1 penetration into the wound and total pixel count were measured by NIS-elements AR software.

***P. aeruginosa* and *S. aureus* planktonic co-culture**

Overnight cultures of PAO1 and USA300 or *crtM*::Tn were diluted to OD_{600} 0.05 and combined at a ratio of 1:1 in LB. The co-culture was incubated at 37˚C shaking at 200rpm for 24h. It was serially diluted and plated on MSA to enumerate for *S. aureus* CFUs. *S. aureus* survival was normalized to the CFUs at 0h.

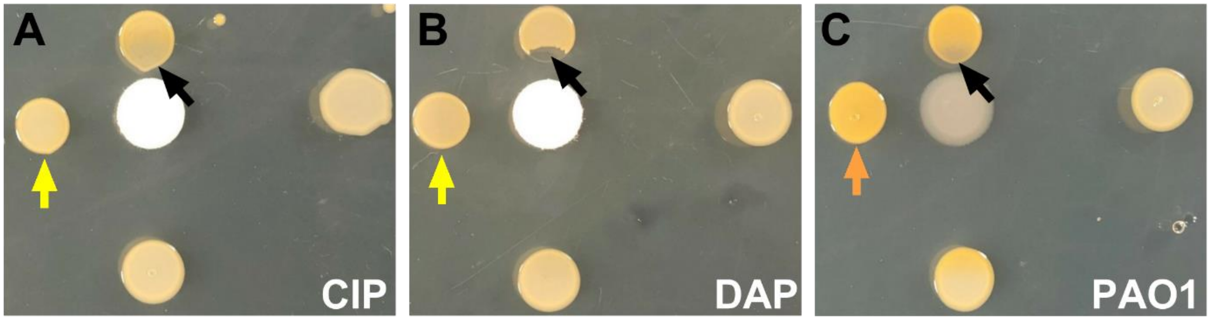

Fig. S1. *S. aureus* STX production is not induced by antibiotic-mediated growth inhibition. USA300 was grown at different distances to discs soaked in 5µL of 1mg/mL ciprofloxacin (CIP), 10mg/mL daptomycin (DAP) or PAO1 overnight culture on solidified media in a macrocolony proximity assay. Yellow arrows point to USA300 colonies with no color change, orange arrows point to USA300 colonies with increased yellow pigmentation and black arrows point to USA300 colonies with inhibited growth.

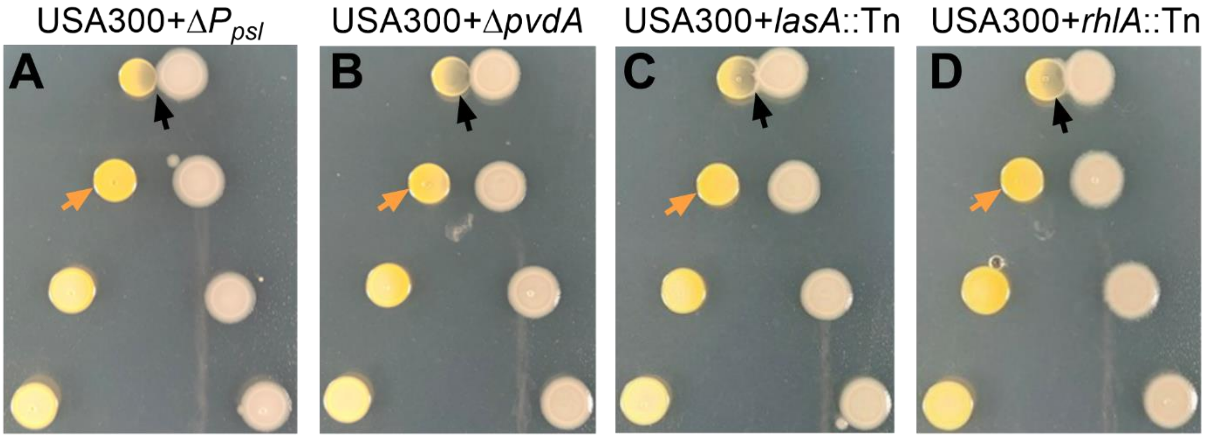

Fig. S2. *S. aureus* STX production when grown with PAO1 mutants with decreased antagonism towards *S. aureus*. USA300 was grown at different distances to PAO1 mutants deficient in producing exopolysaccharide Psl (A, ΔP_{psl}), pyoverdine (B, Δ*pvdA*), protease LasA (C, *lasA*::Tn) or rhamnolipid (D, *rhlA*::Tn) on solidified media in a macrocolony proximity assay. The orange arrows point to USA300 with increased yellow pigmentation, and the black arrows point to USA300 growth inhibition by PAO1.

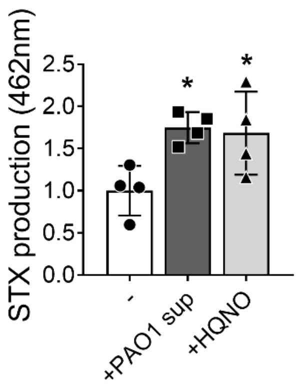

Fig. S3. *P. aeruginosa* HQNO induces *S. aureus* STX production in SCFM2. STX production in SCFM2-grown USA300 treated with or without 5% filter-sterilized PAO1 spent media (sup) or 5μM of HQNO was measured. The results were normalized to the untreated group. Data are presented as mean ± SD from the results of at least 3 biological replicates, each with 2 technical replicates. *, P<0.05, compared to the untreated group, determined by one-way ANOVA.

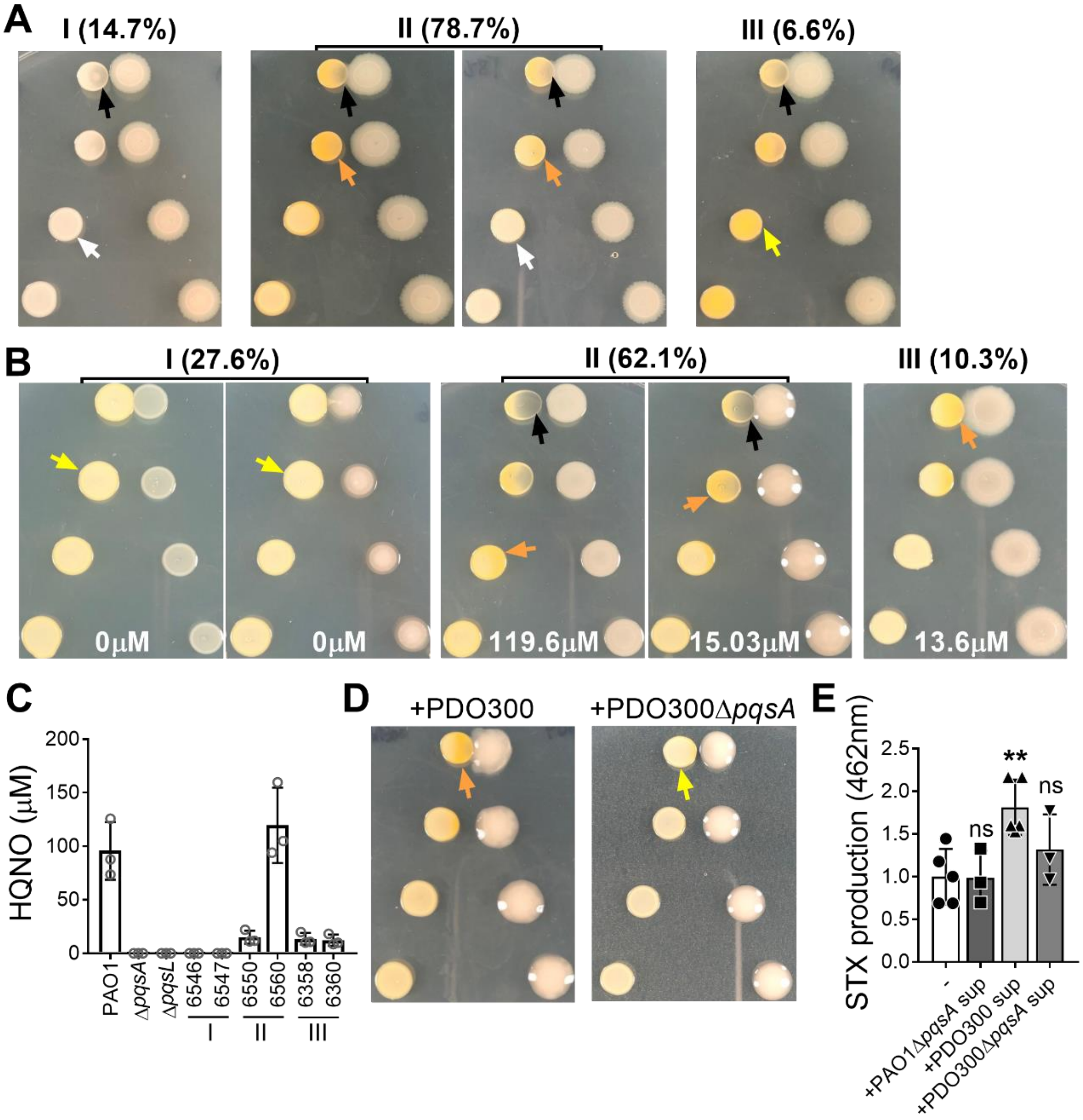

Fig. S4. *P. aeruginosa* induction of *S. aureus* STX production is prevalent among clinical isolates. (A) Representative images and their respective proportions (%) of 3 classes of *S. aureus* clinical isolates when grown with *P. aeruginosa* PAO1 in a macrocolony proximity assay. Class I (14.7%) isolates were white colonies. STX production in Class II (78.7%), despite different intrinsic colors of the colonies (yellow: left; white: right), was induced by adjacent PAO1. Class III (6.6%) had yellow colonies, but no STX induction by PAO1. (B) Representative images and their respective proportions (%) of 3 classes of *P. aeruginosa* clinical isolates when grown with *S. aureus* USA300 in a macrocolony proximity assay. Class I (27.6%) did not induce STX production

in the adjacent USA300 macrocolonies. Class II (62.1%) inhibited USA300 growth and induced STX production. Class III (10.3%) induced STX production in USA300 without growth inhibition. Both mucoid (right) and non-mucoid (left) strains were found in Class I and II. HQNO production of the representative strains was quantified and labeled at the bottom of each image. (C) HQNO production of 2 representative *P. aeruginosa* isolates from each class was measured by LC/MS. (D) USA300 was grown at different distances to mucoid PDO300 (left) or PDO300Δ*pqsA* (right) on solidified media in a macrocolony proximity assay. For A, B and C, the yellow arrows point to *S. aureus* colonies with no color change while the orange arrows depict *S. aureus* colonies with increased yellow pigmentation. The black arrows point to *S. aureus* growth inhibition by *P. aeruginosa*. (E) STX production in USA300, treated with (+) or without (-) 20% filter-sterilized PAO1Δ*pqsA*, PDO300 or PDO300Δ*pqsA* spent media (sup), was measured after methanol extraction at 462nm. The results were normalized to the untreated group. Data are presented as mean ± SD from the results of at least 3 biological replicates, each with 2 technical replicates. **, $P < 0.01$; ns, not significant, compared to the untreated group, determined by one-way ANOVA.

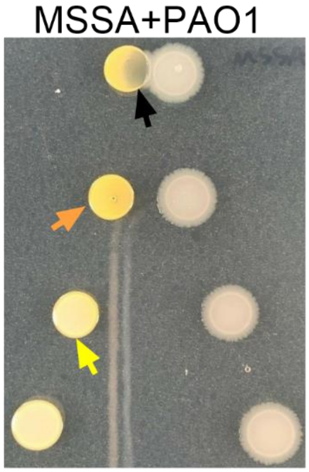

Fig. S5. Methicillin-sensitive *S. aureus* STX production is induced by *P. aeruginosa*. MSSA was grown at different distances to PAO1 on solidified media in a macrocolony proximity assay. The orange arrow points to MSSA with increased yellow pigmentation, the yellow arrow points to MSSA with no color change, and the black arrow points to MSSA growth inhibition by PAO1.

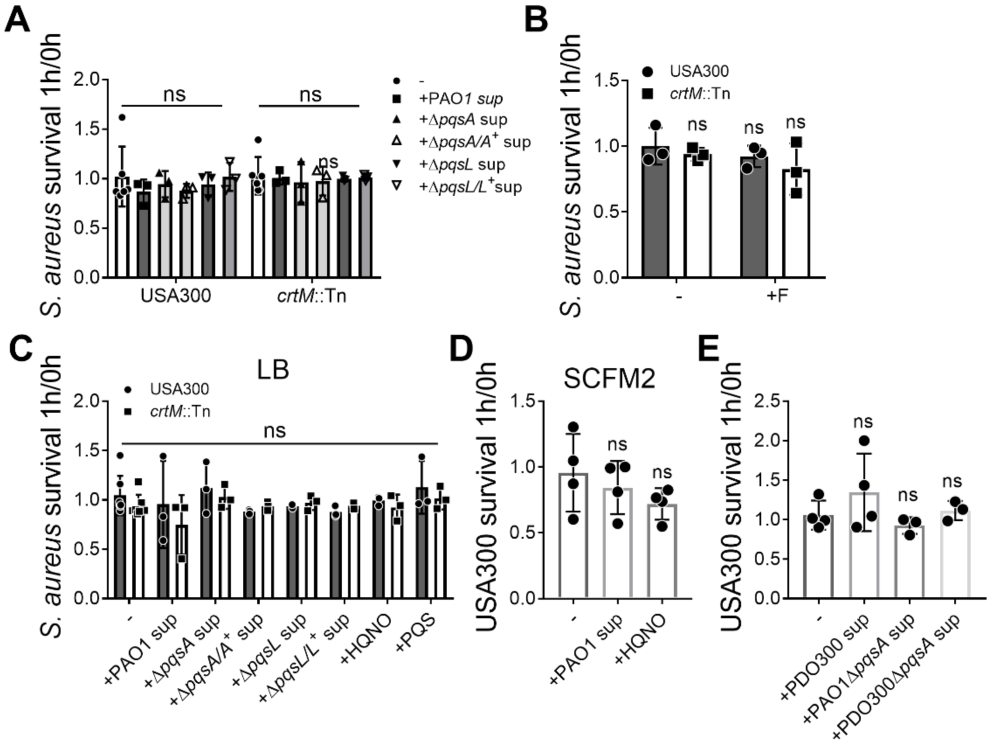

Fig. S6. *S. aureus* survial when treated with H_2O_2. (A) USA300 and *crtM*::Tn were pre-treated with or without 5% filter-sterilized *P. aeruginosa* spent media (sup) overnight and then subjected to 3% H_2O_2 killing for 1h. (B) USA300 and *crtM*::Tn were grown overnight in the presence of 50µg/mL flavone (+F) to inhibit STX production, mixed with an equal amount of PAO1, and subjected to 3% H_2O_2 killing for 1h. (C,D) USA300 and *crtM*::Tn were pre-treated with or without 5% (v/v) filter-sterilized *P. aeruginosa* spent media (sup), or 5µM HQNO or PQS overnight, mixed with an equal amount of PAO1, and subjected to 3% H_2O_2 killing for 1h in either LB (C) or SCFM2 (D). (E) USA300 was pre-treated with or without 20% (v/v) filter sterilized PAO1Δ*pqsA*, PDO300 or PDO300Δ*pqsA* spent media (sup), mixed with an equal amount of PDO300, and subjected to 3% H_2O_2 killing for 1h. *S. aureus* survival is presented as CFUs normalized to the starting CFUs at 0h. Data presented as mean ± SD from the results of at least 3 biological replicates, each with 3 technical replicates. ns, not significant, compared to USA300 with no treatment (-), determined by ANOVA.

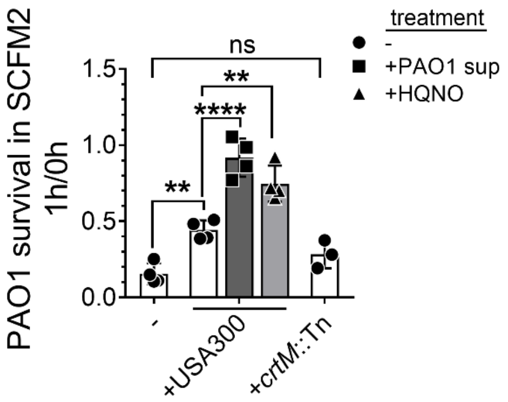

Fig. S7. STX induction protects *P. aeruginosa* from H_2O_2-mediated killing in SCFM2. PAO1, alone or mixed with an equal amount of *S. aureus* with various treatments, was subjected to 3% H_2O_2-mediated killing for 1h in SCFM2. USA300 was pre-treated with or without 5% filter-sterilized PAO1 spent media (sup), or 5μM HQNO overnight. PAO1 survival is presented as CFUs normalized to the starting CFUs at 0h. Data are presented as mean ± SD from the results of at least 3 biological replicates, each with 3 technical replicates. **, $P < 0.01$; ****, $P < 0.0001$; ns, not significant, determined by one-way ANOVA.

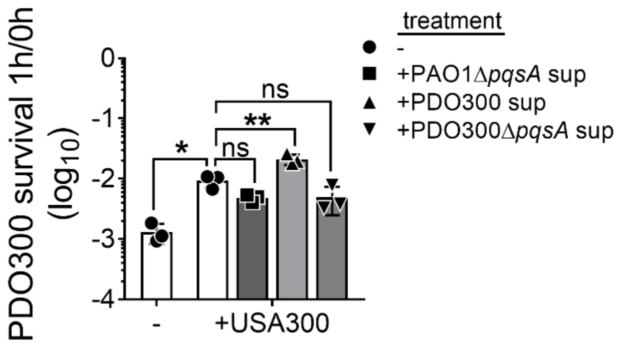

166 Fig. S8. STX induction protects mucoid *P. aeruginosa* from H_2O_2-mediated killing. PDO300, either alone or mixed with an equal amount of USA300 with various treatments, was subjected to 3% H_2O_2-mediated killing for 1h. USA300 was pre-treated with or without 20% (v/v) filter-sterilized PAO1Δ*pqsA*, PDO300 or PDO300Δ*pqsA* spent media (sup). PDO300 survival is presented as CFUs normalized to the starting CFUs at 0h. Data are presented as mean ± SD from the results of at least 3 biological replicates, each with 3 technical replicates. *, $P < 0.05$; **, $P < 0.01$; ns, not significant, determined by one-way ANOVA.

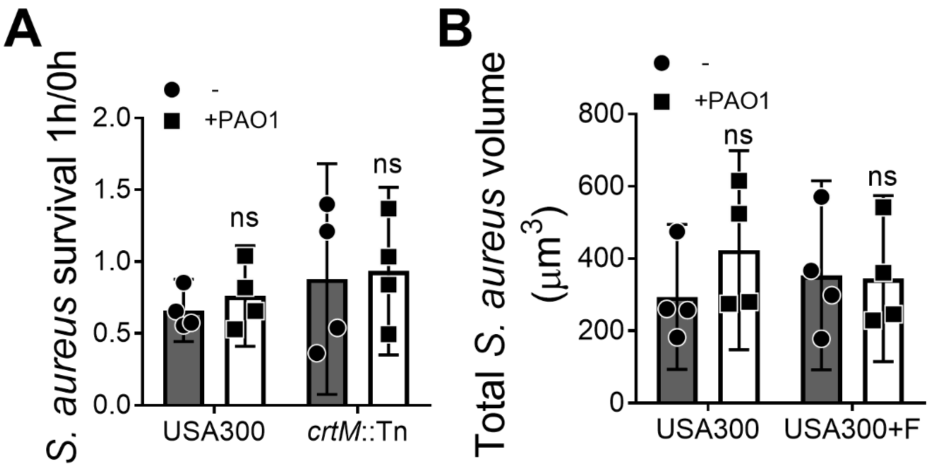

Fig. S9. *S. aureus* survival in the presence of human neutrophils. (A) USA300 or *crtM*::Tn, either alone or mixed with an equal amount of PAO1, was subjected to human neutrophil killing for 1h (MOI = 10 for each species). *S. aureus* survival is presented as CFUs normalized to the starting CFUs at 0h. Data presented as mean ± 95%CI from the results of at 4 biological replicates, each with 3 technical replicates. (B) USA300-GFP was pre-treated with 50μg/mL flavone (+F) to inhibit STX production, then mixed with or without an equal amount of PAO1-TdTomato, was added to adhered human neutrophil (PMN) for 1h. Total *S. aureus* volume was measured by NIS-Element AR software. Data presented as mean ± 95%CI from the results of at least 4 biological replicates, each with 6 technical replicates. ns, not significant, compared to *S. aureus* without the presence of PAO1 (-), determined by two-way ANOVA.

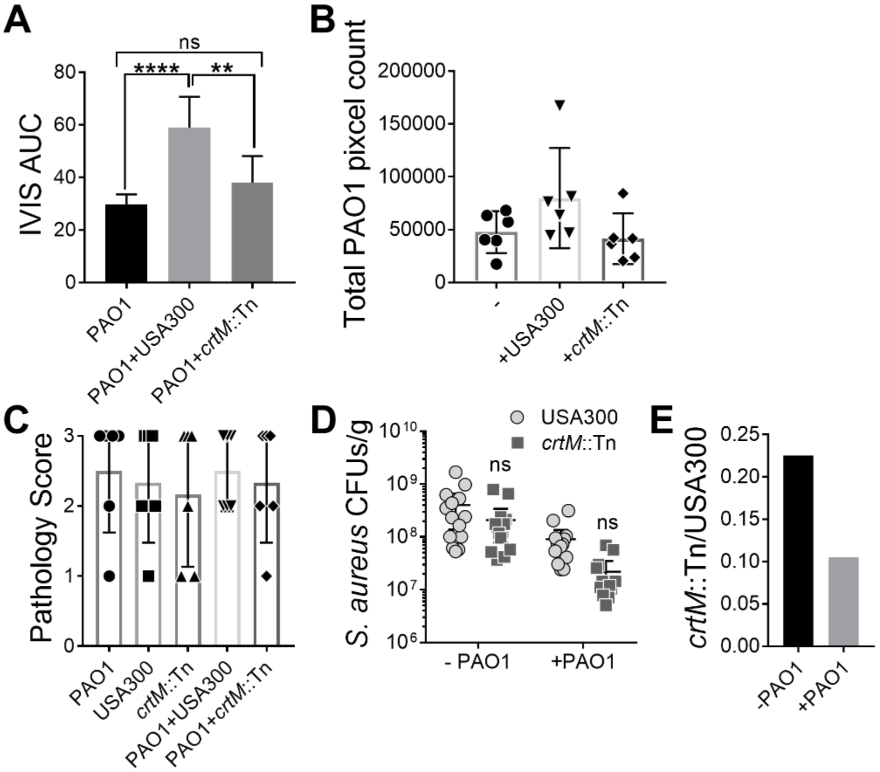

Fig. S10. Analysis of *P. aeruginosa* and *S. aureus* co-infection in the mouse wound model. (A) AUC of Figure 7D comparing PAO1 bioluminescent signal intensity among PAO1 mono-infection and co-infections with USA300 or *crtM*::Tn through the 3-day infection. Data presented as mean ± 95%CI from the results of >12 biological replicates. **, $P < 0.01$; ****, $P < 0.0001$; ns, not significant, determined by one-way ANOVA. (B) PAO1 total pixel count from IF-stained wound sections among all groups was quantified. Data presented as mean ± 95%CI from the results of 6 biological replicates. (C) Pathology scores of the wound tissues 3 days after infection among all groups. Data presented as mean ± 95%CI from the results of 6 biological replicates. (D) USA300 and *crtM*::Tn CFU/g among all groups were quantified. Data presented as mean ± 95%CI from the results of at least 12 biological replicates, each with 3 technical replicates. ns, not significant, compared to USA300 infection, determined by two-way ANOVA. (E) The ratio of *crtM*::Tn survival to that of USA300 was compared between *S. aureus* mono-infection (-PAO1) and co-infection with PAO1 (+PAO1).

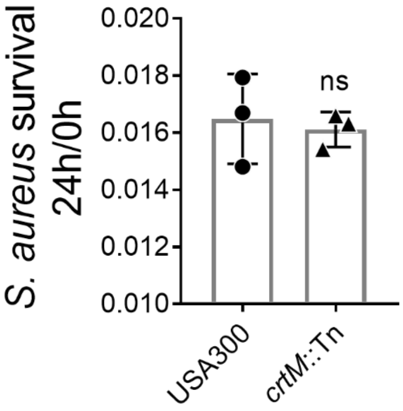

Fig. S11. *S. aureus* survival in co-culture with PAO1. USA300 or *crtM*::Tn were cultured with PAO1 in LB for 24h. *S. aureus* survival was quantified by comparing CFUs at 24h to that of 0h. Data presented as mean ± SD from the results of 3 biological replicates, each with 2 technical replicates. ns, not significant, compared to USA300, determined by one-way ANOVA.

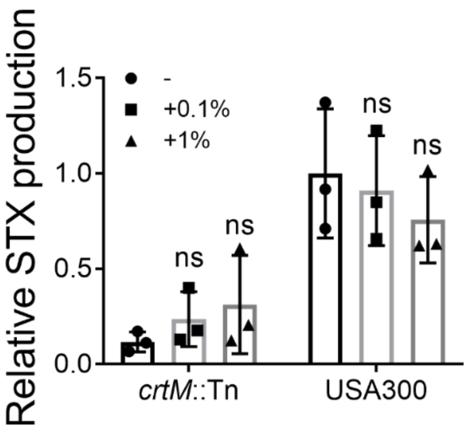

Fig. S12. *S. aureus* STX production is not induced when treated with H_2O_2. STX production in USA300 and *crtM*::Tn, treated with (+) or without (-) 0.1% or 1% H_2O_2, was measured after methanol extraction at 462nm. The results were normalized to the untreated USA300. Data are presented as mean ± SD from the results of 3 biological replicates, each with 2 technical replicates. ns, not significant, compared to the untreated group, determined by one-way ANOVA.

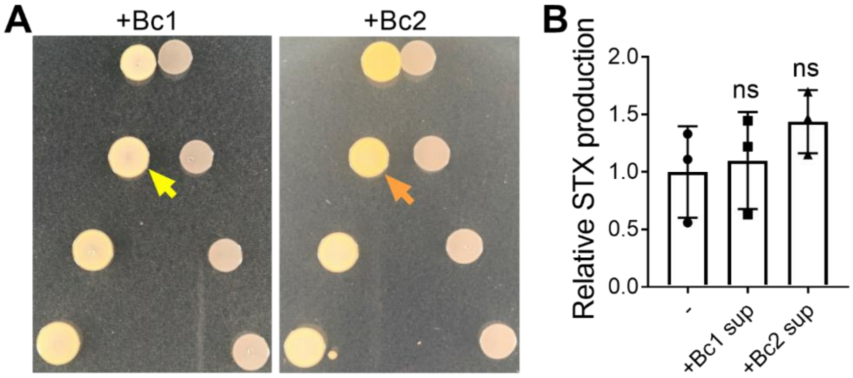

Fig. S13. *S. aureus* STX production can be induced by *B. cepacia* at a modest level. (A) USA300 was grown at different distances to 2 different *B. cepacia* strains (Bc1, Bc2) on solidified media in a macrocolony proximity assay. The orange arrow points to USA300 with increased yellow pigmentation, yellow arrow points to no color change. (B) STX production in USA300, treated with (+) or without (-) filter-sterilized *B. cepacian* spent media (sup), was measured after methanol extraction at 462nm. The results were normalized to the untreated group. Data are presented as mean ± SD from the results of 3 biological replicates, each with 2 technical replicates. ns, not significant, compared to the untreated group, determined by one-way ANOVA.

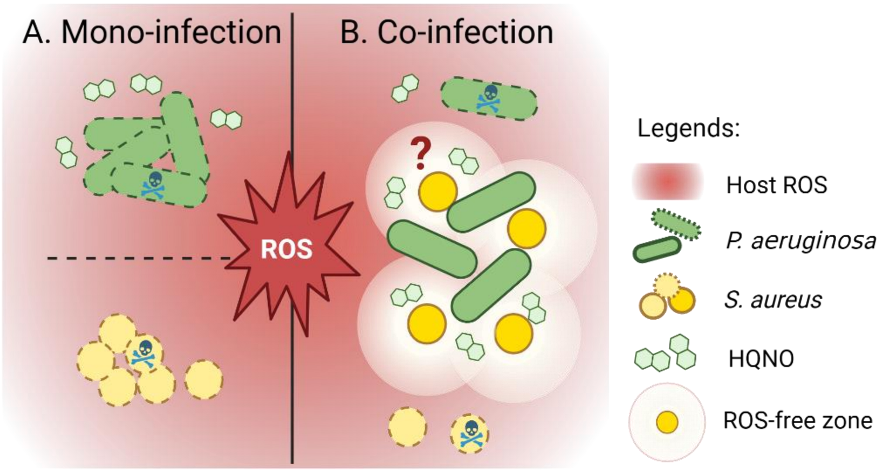

Fig. S14. Schematic summary of *S. aureus* STX-mediated protection to *P. aeruginosa* from host ROS. (A) During mono-infections, *P. aeruginosa* and *S. aureus* are susceptible to host ROS killing indicated by lysed bacteria. (B) During co-infections, *P. aeruginosa*-secreted HQNO induces STX production (indicated by increased yellow pigmentation) in *S. aureus* which protects both bacterial species from host ROS, possibly by creating an ROS-free zone around the *S. aureus* cells. (Figure created with BioRender.com)

Table S1. Strains used in this study

Strains	Description	Source
P. aeruginosa laboratory strains		
PAO1	WT *P. aeruginosa*	(10)
Δ*pqsA*	PAO1 *pqsA* deletion mutant	(11)
Δ*pqsA/A*$^{+}$	Chromosomal complementation of PAO1 Δ*pqsA*	this study
Δ*pqsL*	PAO1 *pqsL* deletion mutant	this study
Δ*pqsL/L*$^{+}$	Chromosomal complementation of PAO1 Δ*pqsL*	this study
PDO300	Mucoid, a *mucA* derivative of PAO1	(12)
PDO300Δ*pqsA*	Mucoid, *pqsA* deleted in PDO300	this study
PAO1-TdTomato	PAO1 carrying a constitutively expressed Td-tomato producing plasmid	(5)
PAO1-lux	Luminescent PAO1	(8)
*Δ*P*psl*	PAO1 *psl* production deficient; *psl* operon promoter deletion mutant	(10)
Δ*pvdA*	PAO1 *pvdA* deletion mutant	(11)
rhlA::Tn	*rhlA* transposon mutant (UWGC:PW6886, PA3479::IS*phoA*/hah)	(13)
lasA::Tn	*lasA* transposon mutant (UWGC:PW4282, PA1871::IS*lacZ*/hah)	(13)
P. aeruginosa clinical isolates		
6546	CF clinical isolate, mucoid	this study
6547	CF clinical isolate	this study
6548	CF clinical isolate	this study
6550	CF clinical isolate, mucoid	this study
6551	CF clinical isolate, mucoid	this study
6559	CF clinical isolate	this study
6560	0CH5M4, CF clinical isolate	(11)
6561	0CH7HJ, CF clinical isolate	(11)
6565	0CHBKC, CF clinical isolate	(11)
6566	0CHBKD, CF clinical isolate	(11)
6354	Wound isolate	this study
6355	Wound isolate	this study
6356	Wound isolate	this study
6357	Wound isolate	this study
6358	Wound isolate	this study
6359	Wound isolate	this study
6360	Wound isolate	this study
6361	Wound isolate	this study
6362	Wound isolate	this study
6363	Wound isolate	this study
6364	Wound isolate	this study
6365	Wound isolate	this study
6366	Wound isolate	this study
6367	Wound isolate	this study

2901	CF clinical isolate	this study
2902	CF clinical isolate, mucoid	this study
2903	CF clinical isolate	this study
2905	CF clinical isolate, mucoid	this study
2906	CF clinical isolate	this study
S. aureus laboratory strains		
USA300	WT *S. aureus*	(14)
MSSA	ATCC 29213, Methicillin sensitive *S. aureus*	ATCC
USA300-GFP	USA300 with constitutively expressed GFP on the chromosome	(6)
crtM::Tn	*crtM* transposon mutant (NE1444, NARSA)	(14)
S. aureus clinical isolates		
6538	CF clinical isolate	this study
6539	CF clinical isolate	this study
6540	CF clinical isolate	this study
6541	CF clinical isolate	this study
6542	CF clinical isolate	this study
6543	CF clinical isolate	this study
6544	CF clinical isolate	this study
6545	CF clinical isolate	this study
6553	CF clinical isolate	this study
6554	CF clinical isolate	this study
6555	CF clinical isolate	this study
6556	CF clinical isolate	this study
6557	CF clinical isolate	this study
6558	CF clinical isolate	this study
6562	CF clinical isolate	this study
6563	CF clinical isolate	this study
6564	CF clinical isolate	this study
6567	CF clinical isolate	this study
6569	CF clinical isolate	this study
6585	CF clinical isolate	this study
6586	CF clinical isolate	this study
6587	CF clinical isolate	this study
6588	CF clinical isolate	this study
6589	CF clinical isolate	this study
6590	CF clinical isolate	this study
6591	CF clinical isolate	this study
6592	CF clinical isolate	this study
6593	CF clinical isolate	this study
6594	CF clinical isolate	this study
6595	CF clinical isolate	this study

6596	CF clinical isolate	this study
6637	CF clinical isolate	this study
4101	Bloodstream isolate	this study
4102	Bloodstream isolate	this study
4103	Bloodstream isolate	this study
4104	Bloodstream isolate	this study
4105	Bloodstream isolate	this study
4106	Bloodstream isolate	this study
4107	Bloodstream isolate	this study
4108	Bloodstream isolate	this study
4109	Bloodstream isolate	this study
4110	Bloodstream isolate	this study
4111	Bloodstream isolate	this study
4112	Bloodstream isolate	this study
4113	Bloodstream isolate	this study
4114	Bloodstream isolate	this study
4115	Bloodstream isolate	this study
4116	Bloodstream isolate	this study
4117	Bloodstream isolate	this study
4118	Bloodstream isolate	this study
4119	Bloodstream isolate	this study
4120	Bloodstream isolate	this study
4121	Bloodstream isolate	this study
4122	Bloodstream isolate	this study
4123	Bloodstream isolate	this study
4124	Bloodstream isolate	this study
4125	Bloodstream isolate	this study
4126	Bloodstream isolate	this study
4127	Bloodstream isolate	this study
4128	Bloodstream isolate	this study
4129	Bloodstream isolate	this study
4130	Bloodstream isolate	this study
B, cepacia strains		
Bc1	*B, cepacia* strain HI2424	(15)
Bc2	*B, cepacia* strain MH1K	(16)

Table S2. STX production and induction in *S. aureus* clinical isolates

	Total #(%)	I	II	III
CF	31 (100%)	5 (16.1%)	23 (74.2%)	3 (9.7%)
Blood	30 (100%)	4 (13.3%)	25 (83.3%)	1 (3.3%)
Total	61 (100%)	9 (14.7%)	48 (78.7%)	4 (6.6%)

I: no STX production
II: STX induced by PAO1
III: produces STX but no induction by PAO1
* No significant difference was found when comparing the classifications of isolates derived from different sources.

Table S3. STX induction by *P. aeruginosa* clinical isolates

	Total #(%)	I	II	III
CF	15 (100%)	7 (46.7%)	8 (53.3%)	0 (0%)
Wound	14 (100%)	1 (7.1%)	10 (71.4%)	3 (21.4%)
Total	29 (100%)	8 (27.6%)	18 (62.1%)	3 (10.3%)

I: no STX induction nor growth inhibition
II: induces STX production with growth inhibition
III: induces STX production without growth inhibition
* No significant difference was found when comparing the classifications of isolates derived from different sources.

SI References

1. Lepine F, Deziel E, Milot S, Rahme LG. 2003. A stable isotope dilution assay for the quantification of the Pseudomonas quinolone signal in Pseudomonas aeruginosa cultures. Biochim Biophys Acta 1622:36-41.
2. Nauseef WM. 2007. Isolation of human neutrophils from venous blood. Methods Mol Biol 412:15-20.
3. Strober W. 2001. Trypan blue exclusion test of cell viability. Curr Protoc Immunol Appendix 3:Appendix 3B.
4. Mishra M, Byrd MS, Sergeant S, Azad AK, Parsek MR, McPhail L, Schlesinger LS, Wozniak DJ. 2012. Pseudomonas aeruginosa Psl polysaccharide reduces neutrophil phagocytosis and the oxidative response by limiting complement-mediated opsonization. Cell Microbiol 14:95-106.
5. Melvin JA, Gaston JR, Phillips SN, Springer MJ, Marshall CW, Shanks RMQ, Bomberger JM. 2017. Pseudomonas aeruginosa Contact-Dependent Growth Inhibition Plays Dual Role in Host-Pathogen Interactions. mSphere 2.
6. Chiu IM, Heesters BA, Ghasemlou N, Von Hehn CA, Zhao F, Tran J, Wainger B, Strominger A, Muralidharan S, Horswill AR, Bubeck Wardenburg J, Hwang SW, Carroll MC, Woolf CJ. 2013. Bacteria activate sensory neurons that modulate pain and inflammation. Nature 501:52-7.
7. Pestrak MJ, Baker P, Dellos-Nolan S, Hill PJ, Passos da Silva D, Silver H, Lacdao I, Raju D, Parsek MR, Wozniak DJ, Howell PL. 2019. Treatment with the Pseudomonas aeruginosa Glycoside Hydrolase PslG Combats Wound Infection by Improving Antibiotic Efficacy and Host Innate Immune Activity. Antimicrob Agents Chemother 63.
8. Lochab V, Jones TH, Dusane DH, Peters CW, Stoodley P, Wozniak DJ, Subramaniam VV, Prakash S. 2020. Ultrastructure imaging of Pseudomonas aeruginosa lawn biofilms and eradication of the tobramycin-resistant variants under in vitro electroceutical treatment. Sci Rep 10:9879.
9. Roy S, Elgharably H, Sinha M, Ganesh K, Chaney S, Mann E, Miller C, Khanna S, Bergdall VK, Powell HM, Cook CH, Gordillo GM, Wozniak DJ, Sen CK. 2014. Mixed-species biofilm compromises wound healing by disrupting epidermal barrier function. J Pathol 233:331-343.
10. Ma L, Jackson KD, Landry RM, Parsek MR, Wozniak DJ. 2006. Analysis of Pseudomonas aeruginosa conditional psl variants reveals roles for the psl polysaccharide in adhesion and maintaining biofilm structure postattachment. J Bacteriol 188:8213-21.
11. Liu Y, Gloag ES, Hill PJ, Parsek MR, Wozniak DJ. 2022. Interbacterial Antagonism Mediated by a Released Polysaccharide. J Bacteriol 204:e0007622.
12. Mathee K, Ciofu O, Sternberg C, Lindum PW, Campbell JIA, Jensen P, Johnsen AH, Givskov M, Ohman DE, Soren M, Hoiby N, Kharazmi A. 1999. Mucoid conversion of Pseudomonas aeruginosa by hydrogen peroxide: a mechanism for virulence activation in the cystic fibrosis lung. Microbiology (Reading) 145 (Pt 6):1349-1357.
13. Jacobs MA, Alwood A, Thaipisuttikul I, Spencer D, Haugen E, Ernst S, Will O, Kaul R, Raymond C, Levy R, Chun-Rong L, Guenthner D, Bovee D, Olson MV, Manoil C. 2003. Comprehensive transposon mutant library of Pseudomonas aeruginosa. Proc Natl Acad Sci U S A 100:14339-44.
14. Fey PD, Endres JL, Yajjala VK, Widhelm TJ, Boissy RJ, Bose JL, Bayles KW. 2013. A genetic resource for rapid and comprehensive phenotype screening of nonessential Staphylococcus aureus genes. mBio 4:e00537-12.
15. Cooper VS, Staples RK, Traverse CC, Ellis CN. 2014. Parallel evolution of small colony variants in Burkholderia cenocepacia biofilms. Genomics 104:447-52.
16. Abdulrahman BA, Khweek AA, Akhter A, Caution K, Tazi M, Hassan H, Zhang Y, Rowland PD, Malhotra S, Aeffner F, Davis IC, Valvano MA, Amer AO. 2013. Depletion of the ubiquitin-

binding adaptor molecule SQSTM1/p62 from macrophages harboring cftr DeltaF508 mutation improves the delivery of Burkholderia cenocepacia to the autophagic machinery. J Biol Chem 288:2049-58.
